# Digital gene expression analysis of NSCLC-patients reveals strong immune pressure, resulting in an immune escape under immunotherapy

**DOI:** 10.1186/s12885-021-09111-w

**Published:** 2022-01-07

**Authors:** Michael Wessolly, Susann Stephan-Falkenau, Anna Streubel, Marcel Wiesweg, Sabrina Borchert, Elena Mairinger, Jens Kollmeier, Henning Reis, Torsten Bauer, Kurt Werner Schmid, Thomas Mairinger, Martin Schuler, Fabian D. Mairinger

**Affiliations:** 1 Institute of Pathology, University Hospital Essen, University of Duisburg-Essen, Hufelandstrasse 55, 45147 Essen, Germany; 2 German Cancer Consortium (DKTK), Partner Site University Hospital Essen, Hufelandstrasse 55, 45147 Essen, Germany; 3 Department of Tissue Diagnostics, Helios Klinikum Emil von Behring, Berlin, Germany; 4 Department of Medical Oncology, West German Cancer Center, University Hospital Essen, Hufelandstrasse 55, 45147 Essen, Germany; 5 Lungenklinik Heckeshorn, Helios Klinikum Emil von Behring, Berlin, Germany; 6 Dr. Senckenberg Institute of Pathology, University Hospital Frankfurt, Goethe University Frankfurt, Frankfurt, Germany

**Keywords:** Massive parallel sequencing, NSCLC, Immunotherapy, Epitope, Processing escape, Deep learning

## Abstract

**Background:**

Immune checkpoint inhibitors (ICIs) are currently one of the most promising therapy options in the field of oncology. Although the first pivotal ICI trial results were published in 2011, few biomarkers exist to predict their therapy outcome. PD-L1 expression and tumor mutational burden (TMB) were proven to be sometimes-unreliable biomarkers. We have previously suggested the analysis of processing escapes, a qualitative measurement of epitope structure alterations under immune system pressure, to provide predictive information on ICI response. Here, we sought to further validate this approach and characterize interactions with different forms of immune pressure.

**Methods:**

We identified a cohort consisting of 48 patients with advanced non-small cell lung cancer (NSCLC) treated with nivolumab as ICI monotherapy. Tumor samples were subjected to targeted amplicon-based sequencing using a panel of 22 cancer-associated genes covering 98 mutational hotspots. Altered antigen processing was predicted by NetChop, and MHC binding verified by NetMHC. The NanoString nCounter® platform was utilized to provide gene expression data of 770 immune-related genes. Patient data from 408 patients with NSCLC were retrieved from The Cancer Genome Atlas (TCGA) as a validation cohort.

**Results:**

The two immune escape mechanisms of PD-L1 expression (TPS score) (*n* = 18) and presence of altered antigen processing (*n* = 10) are mutually non-exclusive and can occur in the same patient (*n* = 6). Both mechanisms have exclusive influence on different genes and pathways, according to differential gene expression analysis and gene set enrichment analysis, respectively. Interestingly, gene expression patterns associated with altered processing were enriched in T cell and NK cell immune activity. Though both mechanisms influence different genes, they are similarly linked to increased immune activity.

**Conclusion:**

Pressure from the immune system will lay the foundations for escape mechanisms, leading to acquisition of resistance under therapy. Both PD-L1 expression and altered antigen processing are induced similarly by pronounced immunoactivity but in different context. The present data help to deepen our understanding of the underlying mechanisms behind those immune escapes.

**Supplementary Information:**

The online version contains supplementary material available at 10.1186/s12885-021-09111-w.

## Introduction

In 2018, 9.6 million people died from cancer or its associated ailments. Based on data from 2015, cancer mortality rates have already surpassed mortality rates from strokes and coronary disease in people below the age of 70 in most western countries. With lung cancer ranking as the deadliest cancer (18,4% of the deaths in 2018) it poses a serious health issue nowadays and in the near future [1–3]. Non-small cell lung cancer (NSCLC) is the most common lung cancer accounting for about 80% of cases. The most prominent subtypes in this category are lung adenocarcinoma (AdC) and lung squamous-cell carcinoma (SCC) [4]. Tobacco exposure is considered the most prominent risk factor for developing lung cancer. It is estimated that 80% of all NSCLC cases are associated with tobacco smoking in various countries including the United States [5].

Recently, immune checkpoint inhibitors (ICIs) have seen frequent use in clinical setups. As a subset of immunotherapy, ICIs are monoclonal antibodies directed against negative regulatory molecules either on immune cells (PD-1 and CTLA-4) or on tumor cells (PD-L1) [6–8]. Though promising results were shown in various clinical studies, the main problem of primary resistance remains. Though significant improvements to clinical benefits have been observed with ICIs, there still remains a large unmet medical need to improve therapy responses [9–11]. Therefore, biomarkers are urgently needed to evaluate patient’s suitability for immunotherapy.

The most frequently discussed biomarker and the only one routinely applied in the clinic is PD-L1 immunohistochemistry. Among available scoring systems, the tumor proportional score (TPS) focusses on PD-L1 expression on tumor cells, while the immune cell (IC) score counts PD-L1 expression on immune cells. Both methods can be summarized with the combined positive score (CPS). Many studies indeed show higher effectiveness of ICIs in patients with high expression levels of PD-L1 (evaluated by TPS scoring) [7, 12–14]. Despite these successes, there are still unexplored caveats concerning PD-L1 expression, some studies displayed equivocal results [15, 16], marking the need for generalized cut-offs regarding the usage of PD-L1 as a biomarker.

NSCLCs are generally considered to have a high rate of somatic mutations compared to other malignancies, thereby increasing the odds of new tumor neoepitopes being generated and presented to immune cells via MHC class I [17–19]. According to neoepitope hypothesis [9, 20, 21] this tumor mutational burden (TMB) is associated with increased tumor immunogenicity and, to a further extend, can serve as a predictive marker for effectiveness of immunotherapy [22–24]. Convincing results suggest that tumors with high TMB respond better to immunotherapy, resulting in the FDA approval of pembrolizumab (a-PD-1 antibody) application in TMB-high solid tumors based on the KEYNOTE-158 study [25, 26]. However, there are still unexplored caveats regarding TMBs usage as a predictive biomarker for immunotherapy. Its clinical utility, particularly in the context of chemo-immunotherapy combinations, has been put into question by several studies [27–31]. In addition, there is no methodological consensus how to define low or high TMB [15, 32, 33]. Though, it should be noted that attempts were made at creating a general applicable assay to determine high and low TMB. Perhaps, the most prominent candidate is the Foundation medicine CDx assay, which was also approved by the FDA and used in the KEYNOTE-158 study [26].

While TMB mainly hints towards the development of potential tumor neoepitopes, it fails to inform which epitopes are generated or if they are bound by the MHC class I complex and could potentially activate immune cells. In previous works we identified altered epitope processing [34, 35] as an important mechanism for tumor immune escape. In particular, those patients characterized by simultaneous PD-L1 expression and high abundance of altered processing showed significantly decreased overall survival.

### Study aim

According to the emerging hallmarks of cancer, tumors need to develop tactics to evade the immune system once it exerts a strong selection pressure towards the tumor [36]. This early pressure can be identified by high expression of genes associated with immune response [37]. In this study, we sought to explore if varying shapes of immune pressure cause the development of different escape mechanisms. This may have deep implications for therapies focused on enhancing the immune response.

## Material and methods

### Demographic data and study design

**Fig. 1:**
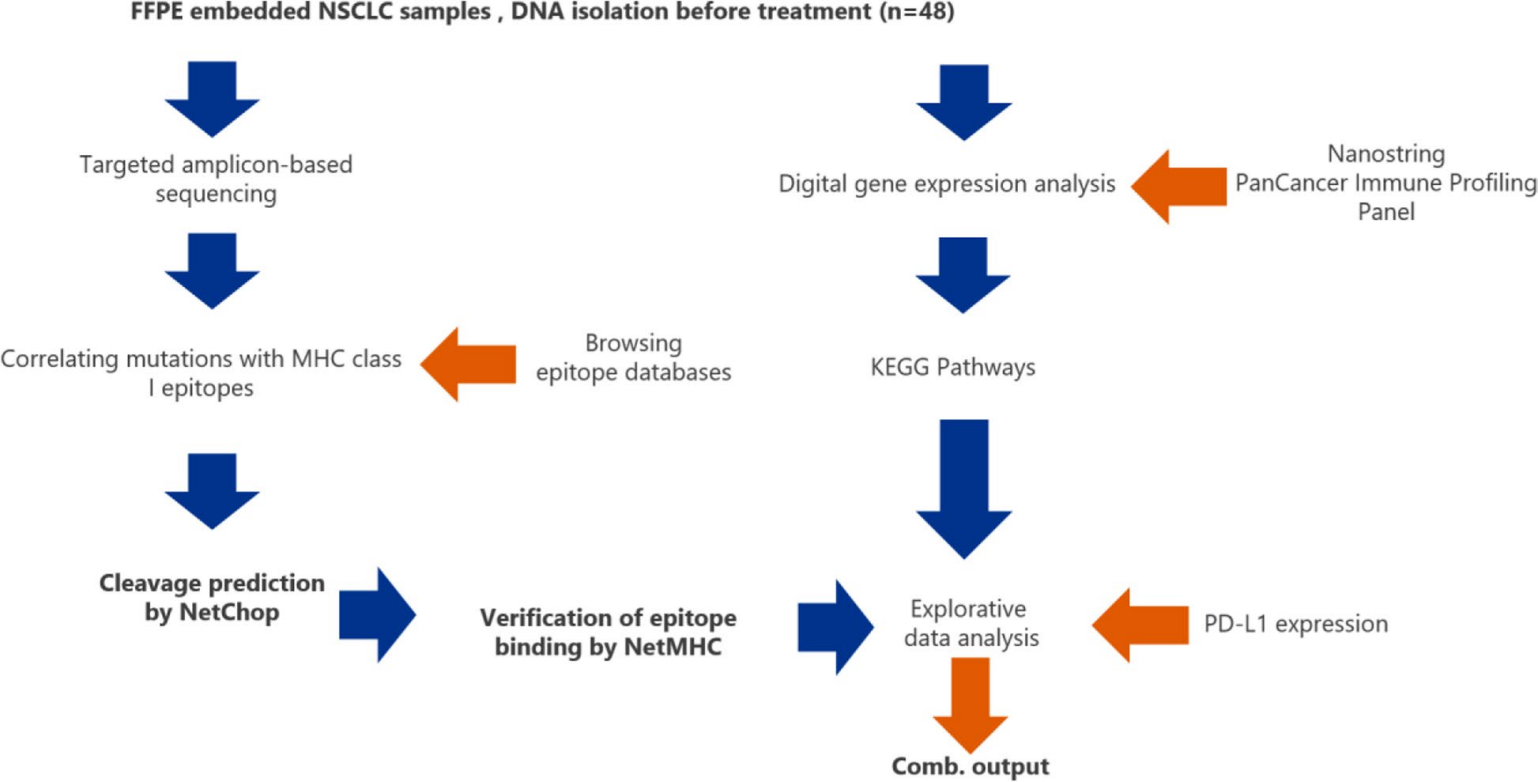
Study design. The figure displays the methodology used within the study procedure

Forty-eight patients were selected for the current study (Fig. 1), they were either diagnosed with advanced/recurrent lung adenocarcinoma (AdC, *n* = 23) or lung squamous-cell carcinoma (SCC, *n* = 25). Diagnostic criteria were based on the World Health Organization (WHO) classification of lung tumors [38]. Between 2012 and 2016, all necessary patient data were collected at the Helios Klinikum Emil von Behring, Berlin, Germany. Patients were included, if sufficient follow-up to estimate overall and progression-free survival, adequate amount of tumor material, was available and biomarkers stratifying for targeted therapy were absent. All patients lacked oncogenic drivers in EGFR, ALK and ROS1. PD-L1 levels were determined by immunohistochemical analysis. The appropriate antibody QR-1 was provided by Quartet, Potsdam, Germany. It has been validated in routine diagnostics to have similar staining activity to E1L3N and 28–8 antibodies. Positive cell detection was defined as membranous stained tumor cells relative to all tumor cells. Tumors were considered PD-L1 positive, if their TPS was at least 1%.

First-line therapy for all patients consisted of chemotherapy. Most patients (45/48) received doublet therapy with one platinum-based component (cis−/carboplatin) and pemetrexed, gemcitabine or vinorelbine. In 28 patients, radiation therapy was applied additionally (Table 1). Each patient received nivolumab (anti-PD-1 antibody) as mono-immunotherapy at least as second-line treatment.

**Table 1:** Overview of patients characteristics

Number of patients	48
Gender	
Male	32
Female	16
Unknown Gender	0
Histological subtype	
Adenocarcinoma	23
Squamous-cell carcinoma	25
Age	
Mean | Median age at diagnosis (years)	64.65 | 64
Range (years)	44–83
OS	
Deceased	34
Alive	14
Range (months)	2.13–78,7
Median | Mean OS (months)	30.78 | 27.27
PFS	
Deceased	35
Alive	13
Range (months)	0.9–31.77
Median | Mean PFS (months)	9.91 | 5.52
RECIST	
Partial response	18
Stable disease	11
Progressive disease	19
PD-L1 status	
TPS > 1%	29
TPS < 1%	13
Treatment before immunotherapy	
Range (previous therapy lines)	2–7
Chemotherapy (first-line)	48
Radiation therapy in addition to chemotherapy	28
ECOG Performance Status	
ECOG = 0	1
ECOG = 1	33
ECOG = 2	12
ECOG > 2	0
Immune-related adverse effects	
Patients affected by irAE	16
Grade 1 irAE	6^a^
Grade 2 irAE	8^a^
Grade 3 irAE	7^a^

^a^Patients can be affected by multiple ailments

In addition, data from 408 NSCLC cases were downloaded from The Cancer Genome Atlas website (TCGA) [39, 40]. Though whole-exome sequencing was performed with these samples, only the genes covered in the targeted panel for the 48 patients mentioned above were considered for analysis. Many patients in this validation cohort were diagnosed with stage I-II NSCLCs, therefore the preferred therapeutic approach consisted of surgical resection and supportive radio-or chemotherapy.

### Nucleic acid preparation

Tumor samples were isolated, fixed with formalin and embedded into paraffin (Formalin-fixed paraffin embedded, FFPE). Based on an eosin and hematoxylin stained slide the tumor area was marked and the amount of tumor cells was determined in the target area. FFPE sections were prepared by using the “Microm HM340E” microtome (Thermo Fisher Scientific). Cut tissue slides were stored at − 20 °C until RNA isolation [3].

Two sections of each FFPE block (10 μm thickness) were used for semi-automatic isolation with the Maxwell purification system (Maxwell RSC RNA FFPE Kit, AS1440, Promega). The purification was performed according to the manufacturer’s instructions. RNA was eluted in 50 μl RNase-free water and stored at − 80 °C.

RNA concentration was measured using a Qubit 2.0 fluorometer (Life Technologies) appertaining the RNA broad-range assay. RNA integrity was assessed using a Fragment Analyzer (Advanced Analytical Inc., Ames, IA, USA) appertaining DNF-489 standard sensitivity RNA analysis kit.

### Digital gene expression analysis

Digital gene expression analysis was performed using the NanoString nCounter® platform (NanoString Technologies, Inc., Seattle, USA) with the NanoString nCounter® PanCancer Immune Profiling Panel. The panel covers 770 genes, which are involved in various immune pathways, including the activation of the innate and adaptive immune response, cell migration and the activity of immune checkpoints, as well as 40 reference genes for biological normalization purposes. Probes were hybridized to 100 ng of total RNA input for 20 h at 65 °C and put into the nCounter® PrepStation. The post-hybridization processing was performed by the nCounter® Max/Flex System using the high-sensitivity protocol and the cartridge was scanned and read on the DigitalAnalyzer at 555 FOV [41].

### NanoString data processing and normalization

NanoString data processing was done with the R statistical programming environment (v4.0.3) [42]. Considering the counts obtained for positive control probe sets, raw NanoString counts for each gene were subjected to a technical factorial normalization, carried out by subtracting the mean counts plus two-times standard deviation from the CodeSet inherent negative controls. Subsequently, a biological normalization using the included mRNA reference genes was performed. Additionally, all counts with *p* > 0.05 after one-sided *t*-test versus negative controls plus 2x standard deviations were interpreted as not expressed to overcome basal noise [41].

### Next generation sequencing and selection of mutations

After DNA isolation on a Maxwell® 16 Research (Promega Corporation, Madison, USA) as recommended in the manufacturer’s protocol, all tumor samples were sequenced using a small panel of 22 genes and 92 amplicons covering hotspots characteristic for NSCLC (Colon Lung v2 AmpliSeq Panel by Thermo Scientific, Waltham, MA, USA).

Non-synonymous mutations with a coverage above 500 reads and an allelic frequency above 3.0% were included into the analysis. Variants with an allelic frequency below 3.0% were filtered out and regarded as artifacts due to formalin fixation. Considering the percentage of tumor cells, the mutations validated needed to be detectable in at least 10% of the tumor sample.

The influence of mutations on proteasomal cleavage was predicted by the machine learning tool NetChop 3.1 [43, 44]. The binding of the resulting epitopes to MHC class I was subsequently simulated by NetMHC 4.0 [45, 46], also based on convolutional neural networks. The whole procedure is described in detail in our previous works [34].

### Explorative data analysis

Explorative data analysis was performed in the R programming environment (v 4.0.3) [42]. The Shapiro-Wilks-test was applied to test for normal distribution of the data [47]. For dichotomous variables either the Wilcoxon Mann-Whitney rank sum test (non-parametric) or two-sided students t-test (parametric) was applied [48]. For ordinal variables with more than two groups, either the Kruskal-Wallis test (non-parametric) or ANOVA (parametric) was used to detect group differences. Double dichotomous contingency tables were analyzed using Fisher’s Exact test. To test dependency of ranked parameters with more than two groups, the Pearson’s Chi-squared test was used. Correlations between metric variables were tested by using the Spearman’s rank correlation test as well as the Pearson’s product moment correlation coefficient for linear modeling. The pathview package can visualize the relation of differentially expressed genes to various signaling pathways. The pathway interactions were provided by the Kyoto Encyclopedia of Genes and Genomes (KEGG) [49]. Significant pathway associations were identified by gene set enrichment analysis using the WEB-based GEne SeT AnaLysis Toolkit (WebGestalt) [50, 51]. Each run was executed with 1000 permutations. The selected database containing pathway information was KEGG. Finally, all associations were ranked according to the false discovery rate (*p* < 0.05).

## Results

### Signs of altered epitope processing were frequently identified in the discovery cohort

Based on the NetChop analysis, around 40% of all identified non-synonymous mutations were associated with changes in proteasomal processing, which also lead to structural changes of presented epitopes. NetMHC analysis revealed that only 11% (of 366 altered epitopes) were still capable to either bind MHC class I molecules in a sufficient manner or trigger an impactful anti-cancer immune response.

### Differential gene expression analysis

To ascertain the possible influence of altered processing on the immune response based on mRNA expression data, patients were categorized by PD-L1 expression on tumor cells (TPS > 1%) and evidence of non-synonymous mutations leading to altered epitope processing. Accordingly, we formed three groups, 1) PD-L1 positive tumors (*n* = 18), 2) tumors with altered epitope processing (*n* = 10), and 3) tumors exhibiting both immune escape mechanisms (*n* = 6) and such showing neither mechanism (*n* = 14).

**Fig. 2:**
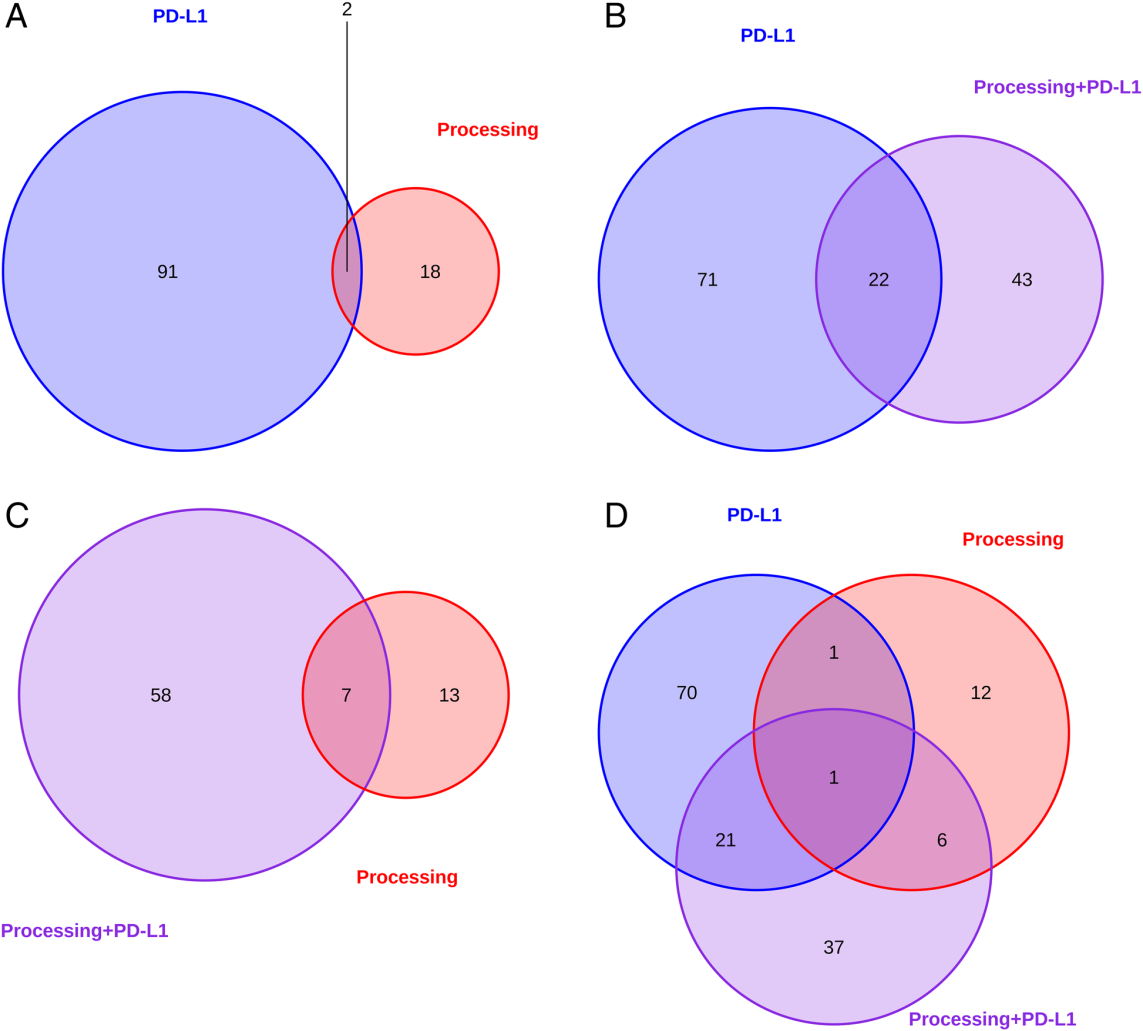
Comparison of differentially expressed genes depending on the escape mechanism. Genes displaying significant expression differences (p < 0.05) in association with a certain mechanism are visualized. Furthermore, overlaps in gene expression are also shown. **A** Significantly expressed genes in correlation with the PD-L1 overexpression (blue) and altered processing (red) are shown. **B** Significantly expressed genes in correlation with PD-L1 overexpression (blue) and both mechanisms (violet, PD-L1 overexpression and altered processing) are shown. **C** Significantly expressed genes in correlation with altered processing (red) and both mechanisms (violet) are shown. **D** Significantly expressed genes between all compared groups (PD-L1 overexpression, altered processing, both mechanisms) are shown

The differentially expressed genes varied strongly between tumors showing an immune escape based on altered epitope processing and those showing PD-L1 expression. 98% and 90% of all differentially expressed genes were specific for each group, respectively (supplemental Figs. 1 and 2 and Fig. 2A). Interestingly, tumors exhibiting both immune escape mechanisms presented a similar gene expression pattern as PD-L1 positive tumors (supplemental Fig. 3, Fig. 2B). Comparing samples with only one mechanism to those presenting both mechanisms, 24% and 35% of genes showed similar expression patterns. 37 (56%) additional genes, which are not shared with the single positive groups, showed specific overexpression in the double-positive group (supplemental Figs. 1, 2 and 3, Fig. 2D).

In the validation cohort, 33% (AdCs) and 62% (SCCs) of all non-synonymous mutations were associated with altered processing. Altered processing significantly affects expression of more genes compared to the discovery cohort (*n* = 161 vs 20). Still, 45% of their respective genes were overlapping (supplemental Figs. 4 and 16).

### Gene set enrichment analysis

**Fig. 3:**
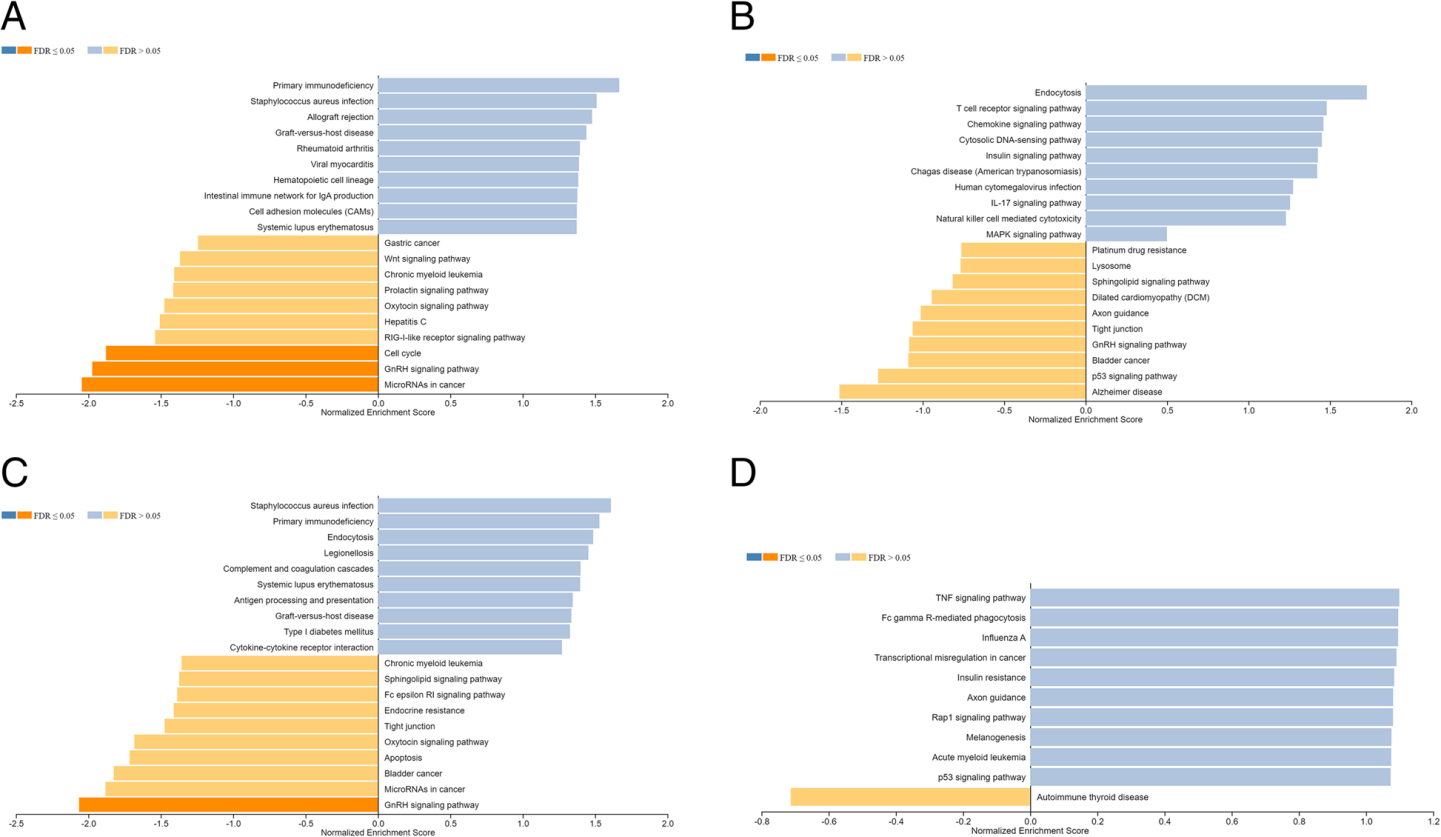
Mechanism-dependant gene set enrichment analysis (GSEA). The analysis shows the enrichment of differentially expressed genes in association with a certain patient group/escape mechanism within a specific biological process. Blue: Strong pathway enrichment in association with a certain immune escape mechanism, orange: Strong pathway enrichment if the escape mechanism is not present. Stronger colouring hints towards significantly increased/reduced gene enrichment in a specific pathway (FDR, *p* < 0.05). **A** Gene set enrichment analysis of patients affected by PD-L1 overexpression. **B** Gene set enrichment analysis of patients affected by altered epitope processing (discovery cohort). **C** Gene set enrichment analysis of patients affected by both PD-L1 overexpression and altered epitope processing (combined). **D** Gene set enrichment analysis of patients affected by altered epitope processing (validation cohort)

Gene set enrichment analysis was performed among all three described patient groups in the discovery (Fig. 3A, B, C, supplemental Figs. 5, 6 and 8) and validation cohort (Fig. 3D, supplemental Fig. 7). Gene expression in association with the pathways “MicroRNAs in cancer”, “GnRH signaling pathway” and “Cell cycle” was significantly decreased in patients displaying PD-L1 expression (*p* < 0.05) (Fig. 3A, supplemental Fig. 5), while genes in association with “Primary immunodeficiency”, “*Staphylococcus aureus* infection” and “Systematic lupus erythematosus” were enriched. “Bladder Cancer”, “GnRH signaling”, “Tight junction interaction”, and “Sphingolipid signaling” were decreased in association with the occurrence of altered processing (Fig. 3B, supplemental Fig. 6). In contrast, “Endocytosis” and more interestingly the immune pathways “T cell receptor signaling”, “IL-17 signaling”, and “Natural killer cell mediated cytotoxicity” were enriched in association with altered epitope processing. Regarding increased expression, the combined patient group showed an enrichment in “Primary immunodeficiency”, “*Staphylococcus aureus* infection”, “Systematic lupus erythematosus” (overlap with PD-L1 expression, Fig. 3A, C, supplemental Figs. 5 and 8), and “Endocytosis” (Overlap with altered processing, Fig. 3B, C, supplemental Figs. 6 and 8). Regarding decreased expression, “GnRH signaling pathway” and “MicroRNAs in cancer” overlapped with the PD-L1 group, while “Tight junction”, “Sphingolipid signaling pathway”, and “Bladder cancer” overlapped with altered epitope processing. In addition to that, the combined group was uniquely associated with increased expression in the following pathways: “Complement system and coagulation cascades”, “Cytokine-cytokine receptor interaction”, and “Antigen processing and presentation”. Strikingly, this patient group also displayed less signs of “Apoptosis” (Fig. 3C, supplemental Fig. 8).

### Various immunological pathways show signs of upregulation in all patient groups

**Fig. 4:**
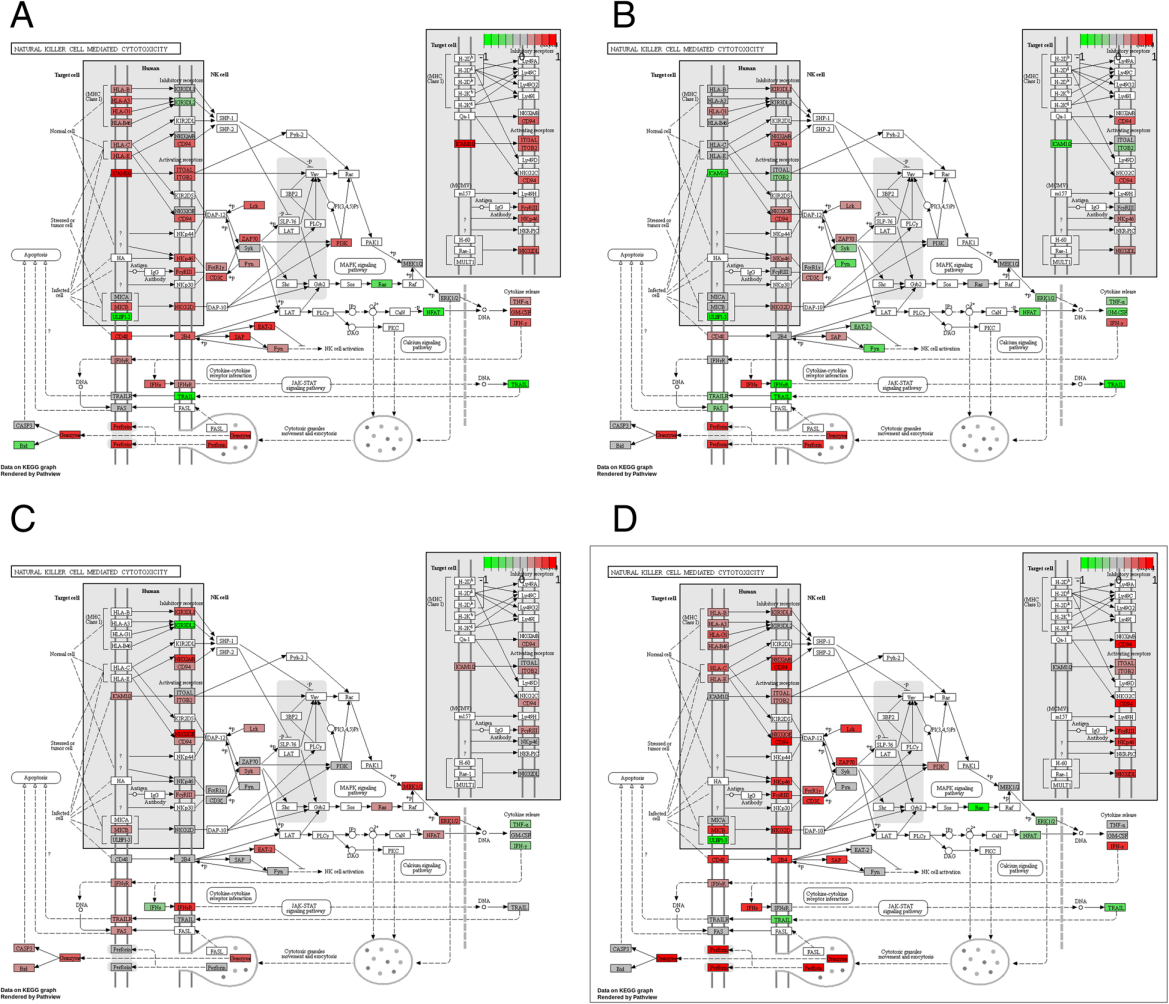
Differential gene expression in natural killer cell mediated cytotoxicity. The plots were created via the pathview package in R. Red: Genes are expressed in association with a specific escape mechanism. Green: Genes are expressed without an escape mechanism being present. Grey: Genes are expressed indifferent of any escape mechanism. **A** KEGG pathway analysis of natural killer cell mediated cytotoxicity in patient expressing PD-L1. **B** KEGG pathway analysis of natural killer cell mediated cytotoxicity in patient showing signs of altered epitope processing (discovery cohort). **C** KEGG pathway analysis of natural killer cell mediated cytotoxicity in patients showing signs of altered epitope processing (validation cohort). **D** KEGG pathway analysis of natural killer cell mediated cytotoxicity in patients showing signs of altered epitope processing and PD-L1 expression

**Fig. 5:**
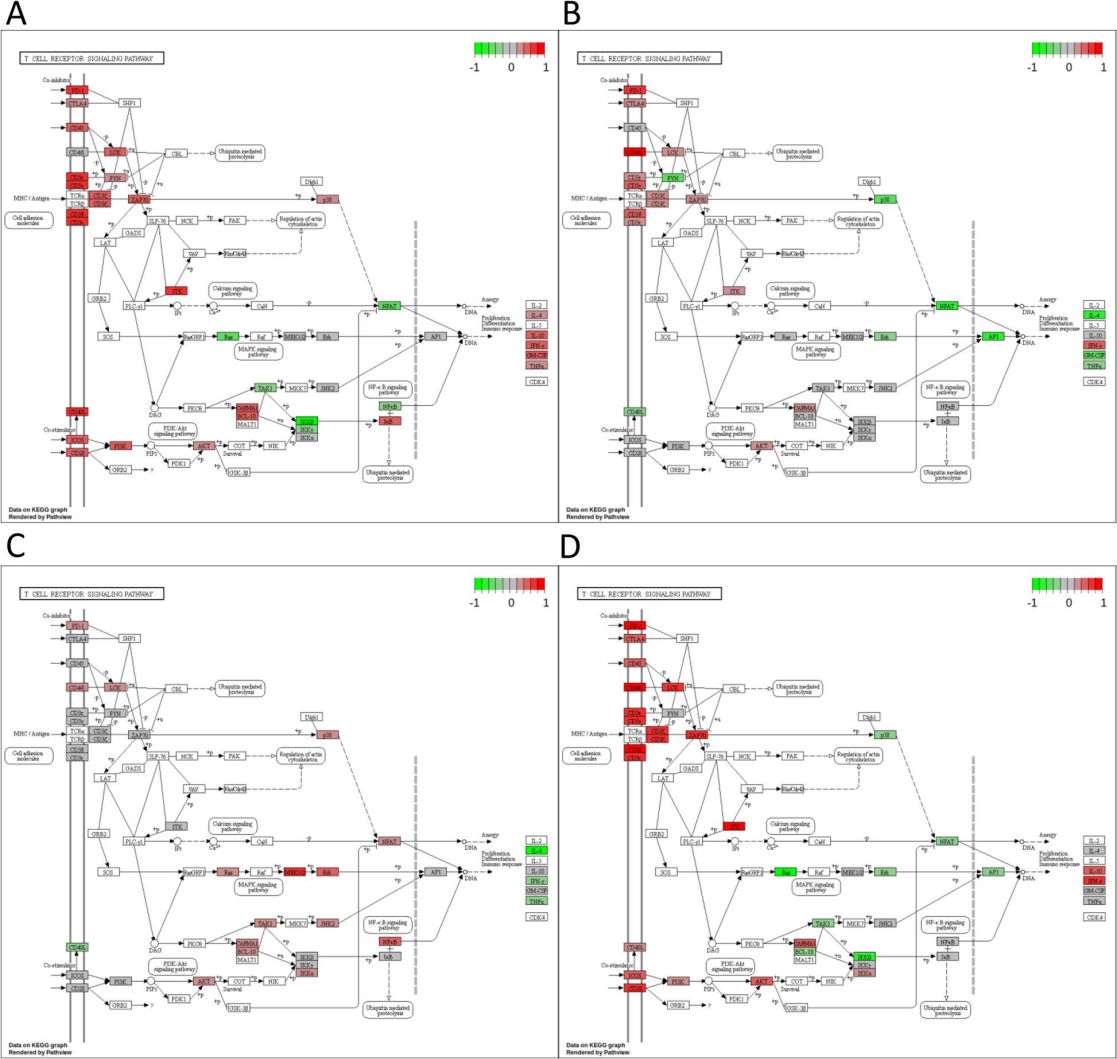
Differential expression of genes in association with T cell receptor signaling. The plots were created via the pathview package in R. Red: Genes are expressed in association with a specific escape mechanism. Green: Genes are expressed without an escape mechanism being present. Grey: Genes are expressed indifferent of any escape mechanism. **A** KEGG pathway analysis of T cell receptor signaling in patients expressing PD-L1. **B** KEGG pathway analysis of T cell receptor signaling in patients showing signs of altered epitope processing (discovery cohort). **C** KEGG pathway analysis of T cell receptor signaling in patients showing signs of altered epitope processing (validation cohort). **D** KEGG pathway analysis of T cell receptor signaling in patients showing signs of altered epitope processing and high levels of PD-L1 expression

We primarily observed changes in three immunological pathways which are considered important for anti-tumor immune activity: T cell receptor signaling (Fig. 5), natural killer cell mediated cytotoxicity (Fig. 4) and signaling of T helper cells (types 1 and 2, supplemental Figs. 9, 10, 11 and 12). Tumors with PD-L1 expression as well as those featuring processing escapes exhibited signs of increased gene expression in all three pathways prior to treatment. PD-L1 expression is seemingly associated with upregulated gene expression within those immunological signaling pathways (Figs. 4A and 5A and supplemental Fig. 9). In contrast, the pathway-associated gene expression was also upregulated in patients displaying altered processing (Figs. 4B and 5B and supplemental Fig. 10), but not as prominent. In the validation cohort altered processing was also linked to increased gene expression in those pathways (Figs. 4C and 5C, supplemental Fig. 11). If patients showed signs of both mechanisms, pathway-associated genes were even more highly upregulated (Figs. 4D and 5D and supplemental Fig. 12).

## Discussion

Before discussing the main conclusions that could be drawn from our work, the small group sizes need to be outlined. Altered processing occurred only in 10 patients, while PD-L1 overexpression was present in 18 patients. Both mechanisms are present in 6 patients. The compared subgroups are rather imbalanced regarding their size. Therefore, further conclusions need to be drawn carefully.

Just by observing the gene expression profiles of each group they are mostly different from each other (Fig. 2 and supplemental Figs. 1, 2, 3 and 4). Despite the differences outlined by differential gene expression analysis, all groups are seemingly related to overexpression of genes in various immunological pathways. The gene expression associated with natural killer cell mediated immune response, T cell receptor signaling and T helper cell type 1 and type 2 signaling is increased in patients with PD-L1 overexpression or altered processing. The combined group (Figs. 4D and 5D, supplemental Fig. 12) indicates that both mechanisms are similarly induced by strong gene expression in these immunological pathways. However, comparatively less genes are expressed in patients with altered processing (NK cell signaling: 43 vs 23, T cell signaling: 27 vs 15) than in patient with PD-L1 overexpression (Figs. 4A, B and 5A, B, supplemental Figs. 9 and 10).

The patient groups were correlated to the immune signature proclaimed by Ayers, Ribas and McClanahan in 2017 [37]. This signature is linked to T cell-associated inflammation, which leads to interferon gamma secretion (IFNγ) and furthermore to increased PD-L1 expression. The patient group, displaying both escape mechanisms apparently has the strongest correlation to the immune signature (supplemental Figs. 14 and 15). One caveat of this finding is again the above-mentioned low group size. Furthermore, despite the correlation of the combined group being close to significant (*p* = 0.05), the data points of this group and the group with PD-L1 overexpression strongly overlap. This could be a hint that PD-L1 overexpression exerts the bigger influence within the combined group. However, PD-L1 expression alone does not display the strongest correlation with the immune signature. Only by including the group displaying altered processing they score the highest. This might give us a further hint that when faced with a strong immune response, it might be effective for the tumor to select both mechanisms for an effective immune escape.

PD-L1 is often expressed on somatic cells, in order to regulate an overshooting immune response. Furthermore, increased PD-L1 expression also serves as an immune escape mechanism for tumors, since it allows them to shut down an immune response [10, 11]. However, this also allows PD-L1 to be used as a predictive biomarker for anti-PD-1/PD-L1 immunotherapy [12], which itself counters the downregulation of the immune response induced by PD-L1. Altered epitope processing has previously been investigated by our group [34, 35, 52] and it was suggested as an additional immune escape mechanism, termed processing escape (supplemental Fig. 13). Processing escapes can mechanistically work in two ways. First, the epitope sequences are prolonged/shortened because mutations change the cleavage patterns of the proteasome. As a consequence, they are unsuitable for MHC binding and are no longer presented on the cell surface. Other research has shown that immune dominant epitopes can be removed from the epitope repertoire by a process called immune editing [9, 53, 54]. Second, the epitopes are still capable of successful MHC class I binding but are less effective in activating T cells due to their altered form.

We assume differences in the temporal aspect of the evolution regarding the two escape mechanisms. PD-L1 expression is an immediate response against pressure by the immune system. It serves as a reactive mechanism by the tumor to induce an immune escape (Figs. 4A and 5A and supplemental Fig. 9). However, it must be noted that PD-L1 expression is not exclusively induced by a direct immune reaction. Therefore, the activity of oncological factors like EGFR, Ras, MAPK, EML4-ALK, MET and PI3K-Akt as well as IFNγ and HIF-1 may also be associated with increased PD-L1 expression. Despite displaying high PD-L1 expression, patients do not necessarily show durable therapy responses upon ICB application [55–57]. This should also be taken into consideration if PD-L1 as a biomarker is concerned. However, this may not fully apply to our study as patients should lack other signs of immune activity as well. Contrarily to this, we found many hints of immune activity based on gene expression analysis. The expression of cytokines, complement factors and factors associated with the complement system, antigen processing and antigen presentation are increased in the risk group (Fig. 3C). Effector molecules of NK cells like granzymes and perforins are also expressed (Fig. 4). T cell receptor activity is high (Fig. 5), while the Ras-MAPK cascade lacks expression if patients show either PD-L1 expression, signs of altered epitope processing or both (Fig. 5A, B, D). These examples point towards an immune activity within the tumor, which makes immune activity as the major driver of PD-L1 expression more likely. This is especially true when considering that patients were negative for oncogenic drivers in EGFR, ALK and ROS1.

Processing escapes, on the other hand, are forced by natural selection, requiring multiple generation cycles. Therefore, they indeed seem like a slow, more adaptive approach to combat the immune response (Figs. 4B and 5B and supplemental Fig. 10).

There are relevant consequences that could be drawn from the study in regards to immune escape mechanisms. PD-L1 is already used as a biomarker, and high PD-L1 expression supports the use of ICI monotherapy. While anti-PD-1/PD-L1 therapy may seem effective at first, the underlying mechanism of processing escapes may lead then to acquired resistance, since both mechanisms are synergistic. It should also be noted that in our study, tumor biopsies were taken before initiation of therapy. Their gene expression signature already shows high immune pressure from the beginning, which leads to the induction of PD-L1 expression and processing escapes later down the line. Therefore, both mechanisms may serve as a combined biomarker already at initial diagnosis.

## Conclusion

Pressure of the immune system during carcinogenesis lays the foundations for escape mechanisms and future resistance to therapy. Despite different genes influencing each respective mechanism (PD-L1 expression and processing escapes), it seems both can be shaped by a strong immune response, which results in simultaneous activation of both and thereby detrimental clinical outcomes as demonstrated in previous works. Identification of the underlying mechanisms of immune silencing may improve patient selection for immunotherapy.

## Supplementary Information

**Additional file 1: Suppl. Figure 1.** Differential gene expression analysis of all patients showing increased PD-L1 expression. The log-fold changes between each state (PD-L1 positive or negative) are plotted against the *p*-value, displaying significant differences in expression between each state. Grey: Differential gene expression does not differ significantly between each state. Red: Top 10 most differentially expressed genes (*p* < 0.01). **Suppl. Figure 2.** Differential gene expression analysis of all patients showing signs of altered epitope processing. The log-fold changes between each state (altered processing is present or not) are plotted against the p-value, displaying significant differences in expression between each state. Grey: Differential gene expression does not differ significantly between each state. Red: Top 10 most differentially expressed genes (*p* < 0.05). **Suppl. Figure 3.** Differential gene expression analysis of all patients showing signs of altered epitope processing in the validation cohort. The log-fold changes between each state (altered processing is present or not) are plotted against the p-value, displaying significant differences in expression between each state. Grey: Differential gene expression does not differ significantly between each state. Red: Top 10 most differentially expressed genes (*p* < 0.01). **Suppl. Figure 4.** Differential gene expression analysis of all patients showing signs both altered epitope processing and PD-L1 expression. The log-fold changes between each state (Both mechanisms are present or not) are plotted against the *p*-value, displaying significant differences in expression between each state. Grey: Differential gene expression does not differ significantly between each state. Red: Top 10 most differentially expressed genes (*p* < 0.01). **Suppl. Figure 5.** To gain further insight into which biological processes are affected by differential gene expression in patients showing PD-L1 expression, a gene set enrichment analysis was performed. The gene ontology (GO) analysis is part of the greater enrichment analysis. The analysis was performed to estimate the correlation between a patient group/escape mechanism and certain biological functions (red chart), cellular components (blue chart) and molecular functions (green chart). **Suppl. Figure 6.** To gain further insight into which biological processes are affected by differential gene expression in patients showing signs of altered epitope processing (discovery cohort), a gene set enrichment analysis was performed. The gene ontology (GO) analysis is part of the greater enrichment analysis. The analysis was performed to estimate the correlation between a patient group/escape mechanism and certain biological functions (red chart), cellular components (blue chart) and molecular functions (green chart). **Suppl. Figure 7.** To gain further insight into which biological processes are affected by differential gene expression in patients showing signs of altered epitope processing (validation cohort), a gene set enrichment analysis was performed. The gene ontology (GO) analysis is part of the greater enrichment analysis. The analysis was performed to estimate the correlation between a patient group/escape mechanism and certain biological functions (red chart), cellular components (blue chart) and molecular functions (green chart). **Suppl. Figure 8.** To gain further insight into which biological processes are affected by differential gene expression in patients displaying both mechanisms (PD-L1 expression and altered epitope processing), a gene set enrichment analysis was performed. The gene ontology (GO) analysis is part of the greater enrichment analysis. The analysis was performed to estimate the correlation between a patient group/escape mechanism and certain biological functions (red chart), cellular components (blue chart) and molecular functions (green chart). **Suppl. Figure 9.** KEGG pathway analysis of T helper cell (subtype 1 and 2) differentiation in patients expressing PD-L1. The plots were created via the pathview package in R. Genes are either strongly expressed (red) or their expression is reduced (green). **Suppl. Figure 10.** KEGG pathway analysis of T helper cell (subtype 1 and 2) differentiation in patients showing signs of altered epitope processing (discovery cohort). The plots were created via the pathview package in R. Genes are either strongly expressed (red) or their expression is reduced (green). **Suppl. Figure 11.** KEGG pathway analysis of T helper cell (subtype 1 and 2) differentiation in patients showing signs of altered epitope processing (validation cohort). The plots were created via the pathview package in R. Genes are either strongly expressed (red) or their expression is reduced (green). **Suppl. Figure 12.** KEGG pathway analysis of T helper cell (subtype 1 and 2) differentiation in patients showing both signs of altered epitope processing and PD-L1 expression. The plots were created via the pathview package in R. Genes are either strongly expressed (red) or their expression is reduced (green). **Suppl. Figure 13.** Activation of cytotoxic lymphocytes by tumor neoepitopes under immune checkpoint therapy. The figure additionally highlights the role of altered epitope processing. Through high mutational load, mutations change proteasomal cleavage patterns leading to structural changes or disruption of the original epitope. Regardless, the immunogenicity of the tumor neoepitopes is lowered. The T cell does not become active and immune checkpoint inhibition is rendered ineffective, since it cannot promote weak or absent signaling. **Suppl. Figure 14.** Expression of IFN gamma associated genes (based on [37]) in association with patients expressing PD-L1 or showing signs of altered processing. FALSE/FALSE: Patients displaying neither mechanism (green), TRUE/FALSE: Patients display signs of altered epitope processing, but no signs of PD-L1 expression (red). FALSE/TRUE: Patients displaying signs of PD-L1 expression, but without any signs of altered epitope processing (blue). TRUE/TRUE: Patients show signs of both mechanisms (violet). NS: Not significant. *p* = 0.05926. **Suppl. Figure 15.** Expression of an extended mRNA based immune signature (based on [37]) in association with patients expressing PD-L1 or showing signs of altered processing. FALSE/FALSE: Patients displaying neither mechanism (green), TRUE/FALSE: Patients display signs of altered epitope processing, but no signs of PD-L1 expression (red). FALSE/TRUE: Patients displaying signs of PD-L1 expression, but without any signs of altered epitope processing (blue). TRUE/TRUE: Patients show signs of both mechanisms (violet). NS: Not significant. *p* = 0.05623. **Suppl. Figure 16.** The discovery cohort (“Sample”, red) and the validation cohort (dark red) were compared regarding gene expression in association with altered epitope processing.

## Data Availability

Data regarding this work will be made available upon a reasonable request to the corresponding author.

## Abbreviations

AdC: Adenocarcinoma
ANOVA: Analysis of variance
AS: Amino acid
COXPH: Cox proportional-hazards model
CTL: Cytotoxic T-lymphocyte
CTLA-4: Cytotoxic T-lymphocyte-associated Protein 4
DDCT: Double dichotomous contingency tables
DNA: Deoxyribonucleic acid
ER: Endoplasmic reticulum
FDR: False discovery rate
FFPE: Formalin-fixed paraffin-embedded tissue
HLA: Human leucocyte antigen
H&E stain: Hematoxylin and eosin stain
ICI: Immune checkpoint inhibitor
IC50: Half maximal inhibitory concentration
IEDB: Immune epitope data base
IFNγ: Interferon gamma
I/O: Immune-Oncology
Mb: Megabase
MHC: Major histocompatibility complex
mRNA: Messenger ribonucleic acid
NGS: Next-Generation Sequencing
NSCLC: Non-small cell lung cancer
OS: Overall survival
PD-1: Programmed cell death protein 1
PD-L1: Programmed cell death 1 ligand 1
SCC: Squamous-cell carcinoma
SNP: Single nucleotide polymorphism
TAP: Transporter associated with antigen processing
TCGA: The Cancer Genome Atlas
TCR: T cell receptor
TMB: Tumor mutational burden
TH1: T helper cell type 1
TH2: T helper cell type 2
UICC: Union internationale contre le cancer
