# Supplementary material for: Digital gene expression analysis of NSCLC-patients reveals strong immune pressure, resulting in an immune escape under immunotherapy
Source: BMC Cancer. 2022 Jan 7;22:46. doi: 10.1186/s12885-021-09111-w (PMC8740040; doi:10.1186/s12885-021-09111-w)
Supplement: Supplementary file 1 — Additional file 1: Suppl. Figure 1. Differential gene expression analysis of all patients showing increased PD-L1 expression. The log-fold changes between each state (PD-L1 positive or negative) are plotted against the p-value, displaying significant differences in expression between each state. Grey: Differential gene expression does not differ significantly between each state. Red: Top 10 most differentially expressed genes (p < 0.01). Suppl. Figure 2. Differential gene expression analysis of all patients showing signs of altered epitope processing. The log-fold changes between each state (altered processing is present or not) are plotted against the p-value, displaying significant differences in expression between each state. Grey: Differential gene expression does not differ significantly between each state. Red: Top 10 most differentially expressed genes (p < 0.05). Suppl. Figure 3. Differential gene expression analysis of all patients showing signs of altered epitope processing in the validation cohort. The log-fold changes between each state (altered processing is present or not) are plotted against the p-value, displaying significant differences in expression between each state. Grey: Differential gene expression does not differ significantly between each state. Red: Top 10 most differentially expressed genes (p < 0.01). Suppl. Figure 4. Differential gene expression analysis of all patients showing signs both altered epitope processing and PD-L1 expression. The log-fold changes between each state (Both mechanisms are present or not) are plotted against the p-value, displaying significant differences in expression between each state. Grey: Differential gene expression does not differ significantly between each state. Red: Top 10 most differentially expressed genes (p < 0.01). Suppl. Figure 5. To gain further insight into which biological processes are affected by differential gene expression in patients showing PD-L1 expression, a gene set enrichment analysis [file 12885_2021_9111_MOESM1_ESM.docx]

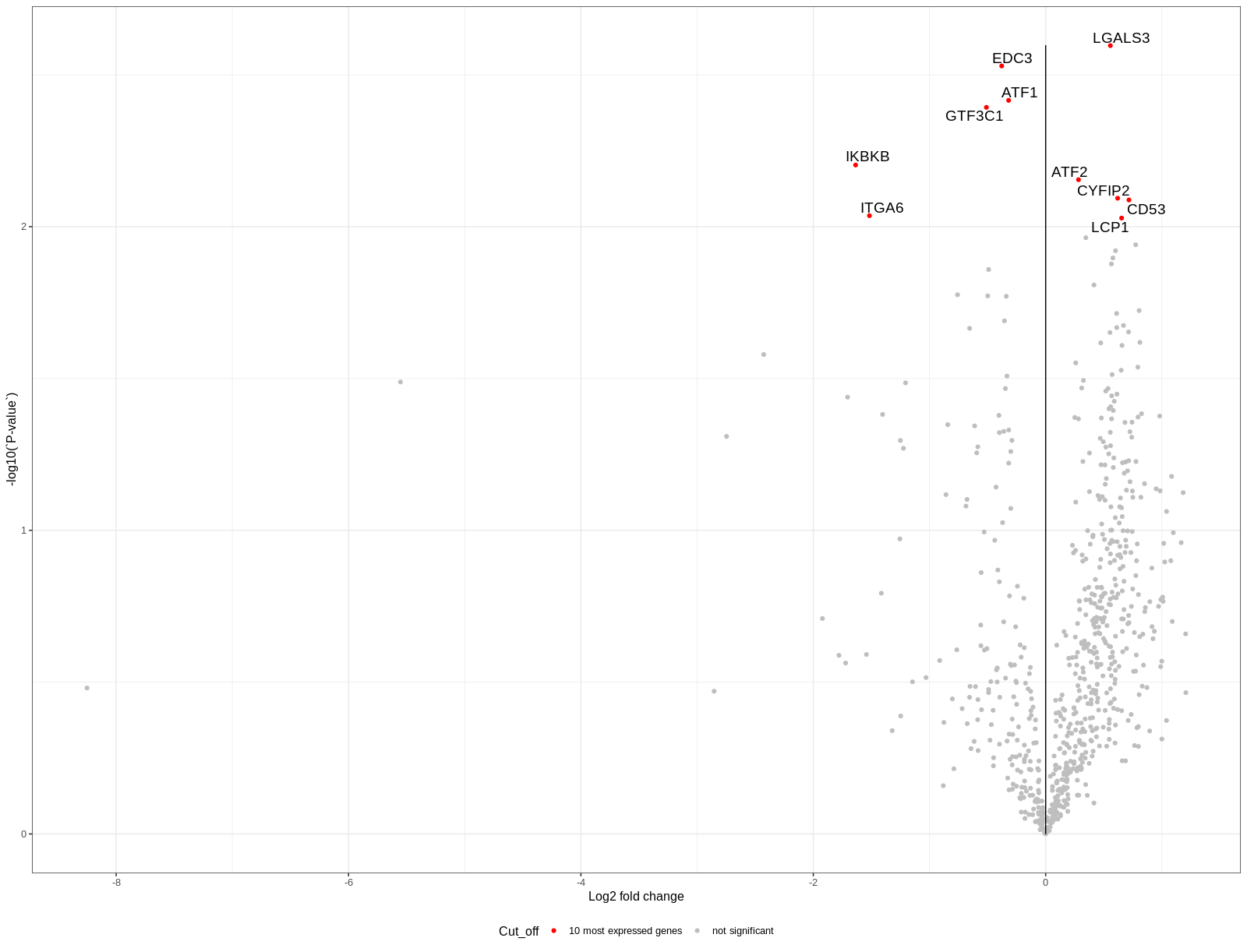


Suppl. Figure 1: Differential gene expression analysis of all patients showing increased PD-L1 expression. The log-fold changes between each state (PD-L1 positive or negative) are plotted against the p-value, displaying significant differences in expression between each state. Grey: Differential gene expression does not differ significantly between each state. Red: Top 10 most differential expressed genes (p < 0.01).


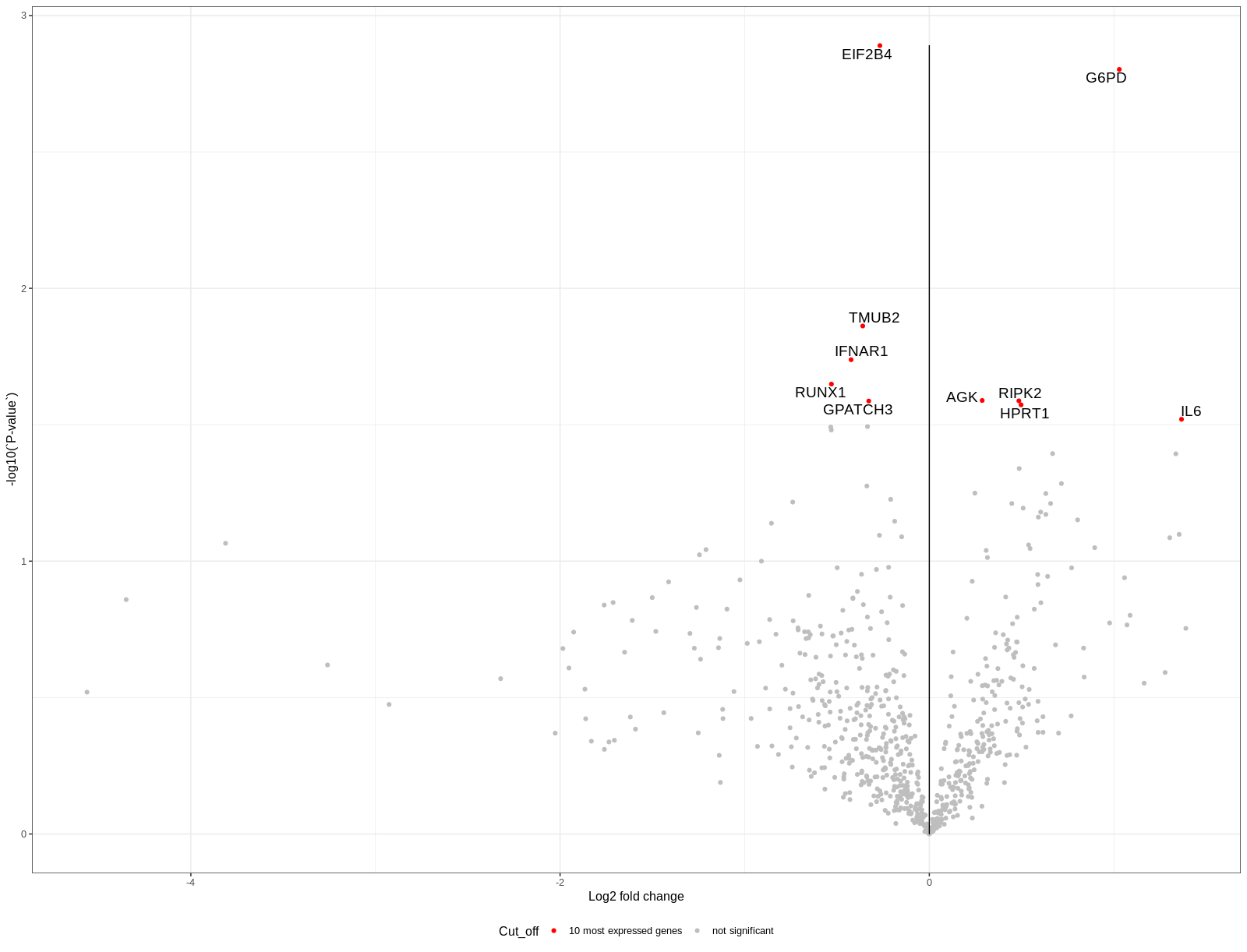


Suppl. Figure 2: Differential gene expression analysis of all patients showing signs of altered epitope processing. The log-fold changes between each state (altered processing is present or not) are plotted against the p-value, displaying significant differences in expression between each state. Grey: Differential gene expression does not differ significantly between each state. Red: Top 10 most differential expressed genes (p < 0.05).


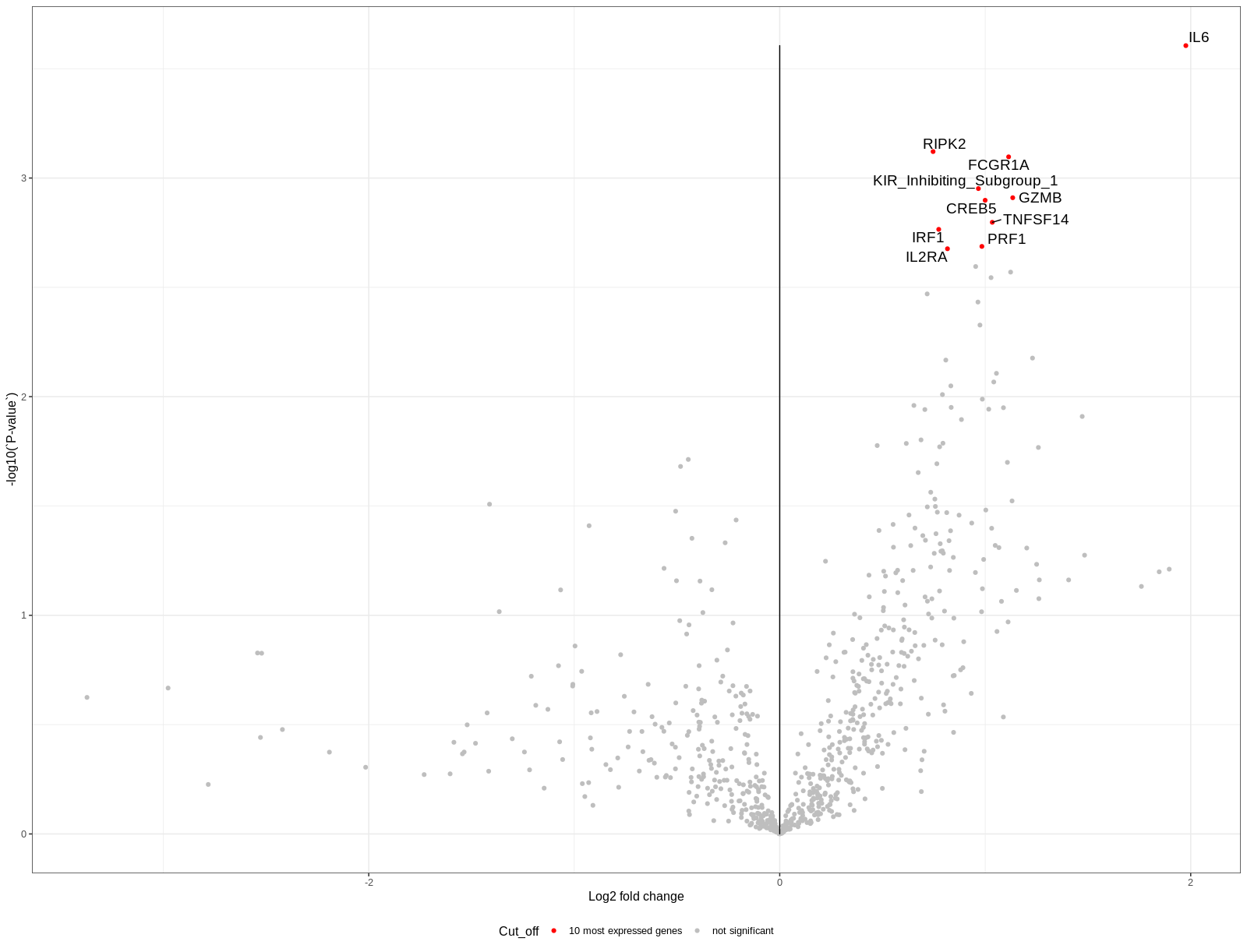


Suppl. Figure 3: Differential gene expression analysis of all patients showing signs of altered epitope processing in the validation cohort. The log-fold changes between each state (altered processing is present or not) are plotted against the p-value, displaying significant differences in expression between each state. Grey: Differential gene expression does not differ significantly between each state. Red: Top 10 most differential expressed genes (p < 0.01).


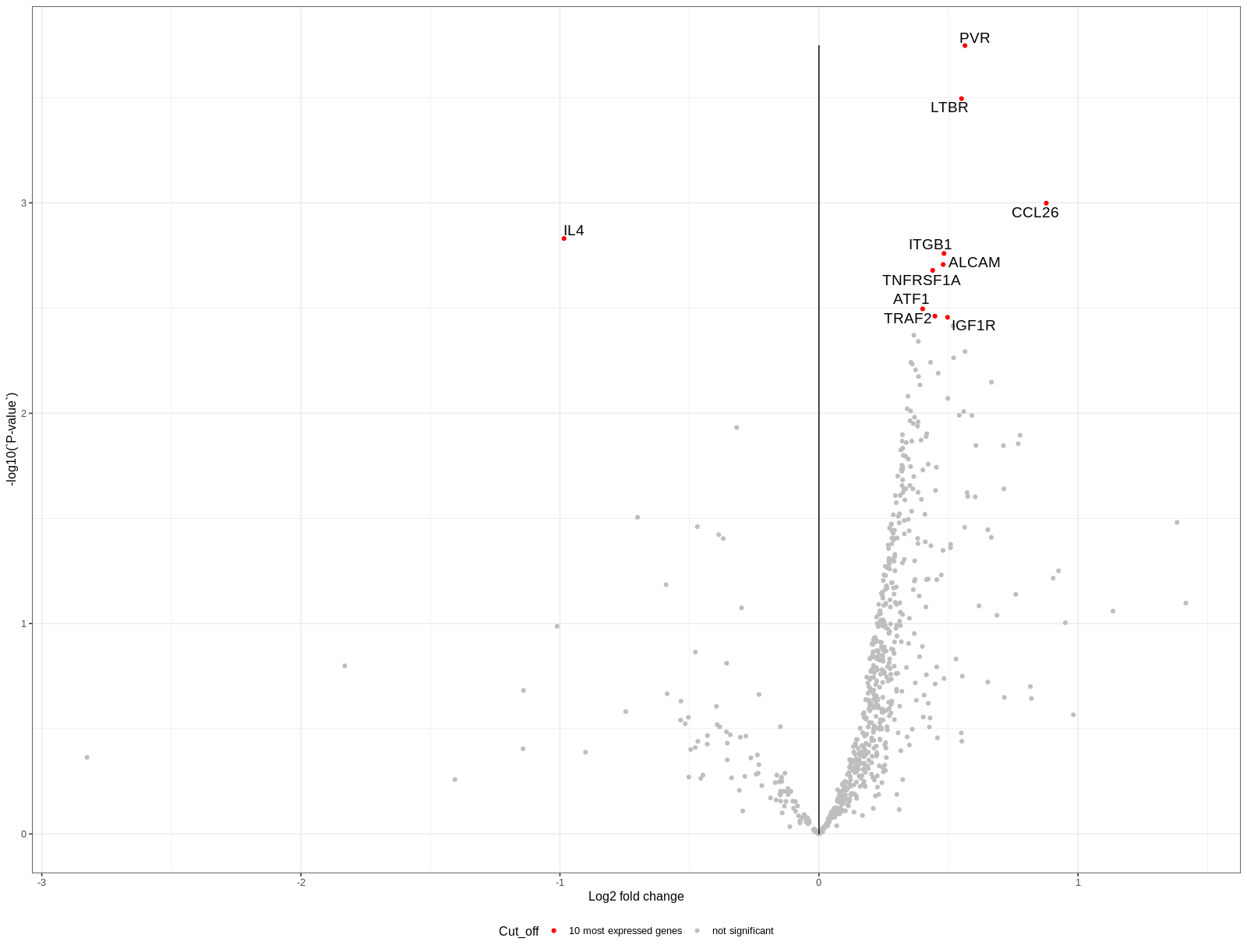


Suppl. Figure 4: Differential gene expression analysis of all patients showing signs both altered epitope processing and PD-L1 expression. The log-fold changes between each state (Both “phenotypes” are present or not) are plotted against the p-value, displaying significant differences in expression between each state. Grey: Differential gene expression does not differ significantly between each state. Red: Top 10 most differential expressed genes (p < 0.01).


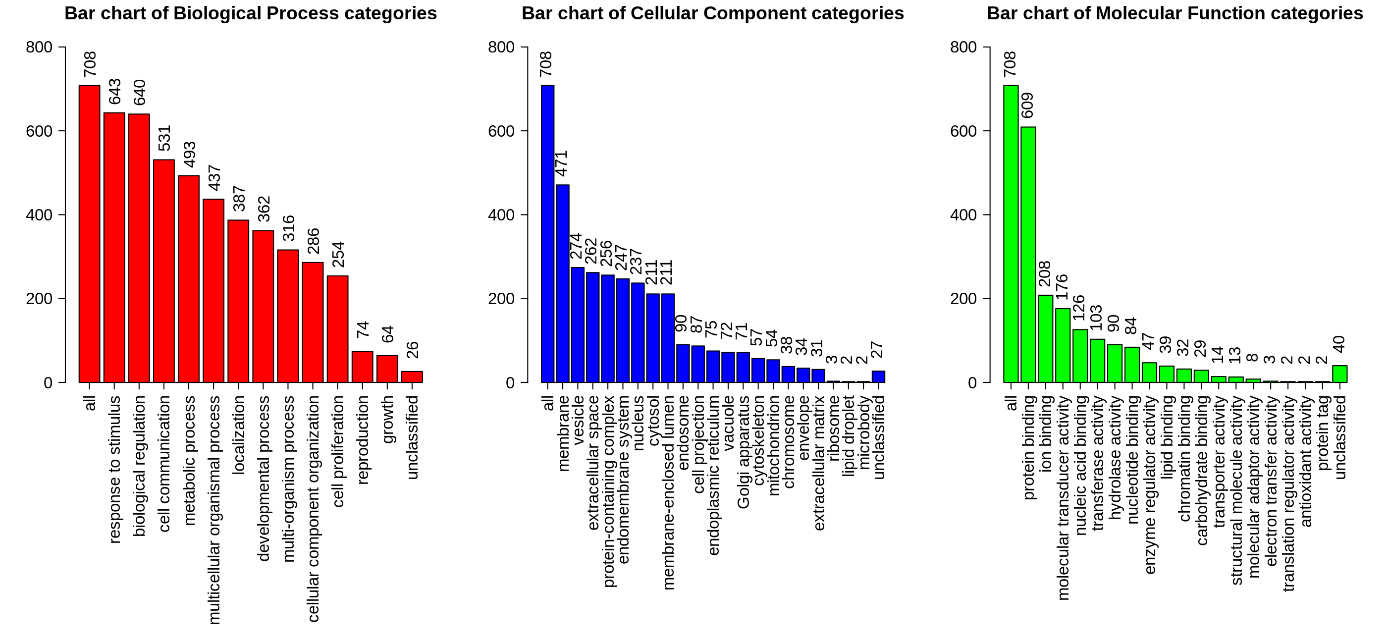


Suppl. Figure 5: To gain further insight into which biological processing are affected by differential gene expression in patients showing PD-L1 expression, a gene set enrichment analysis was performed. The gene ontology (GO) analysis is part of the greater enrichment analysis. The analysis was performed to estimate the correlation between a patient group/escape mechanism and certain biological functions (red chart), cellular components (blue chart) and molecular functions (green chart).


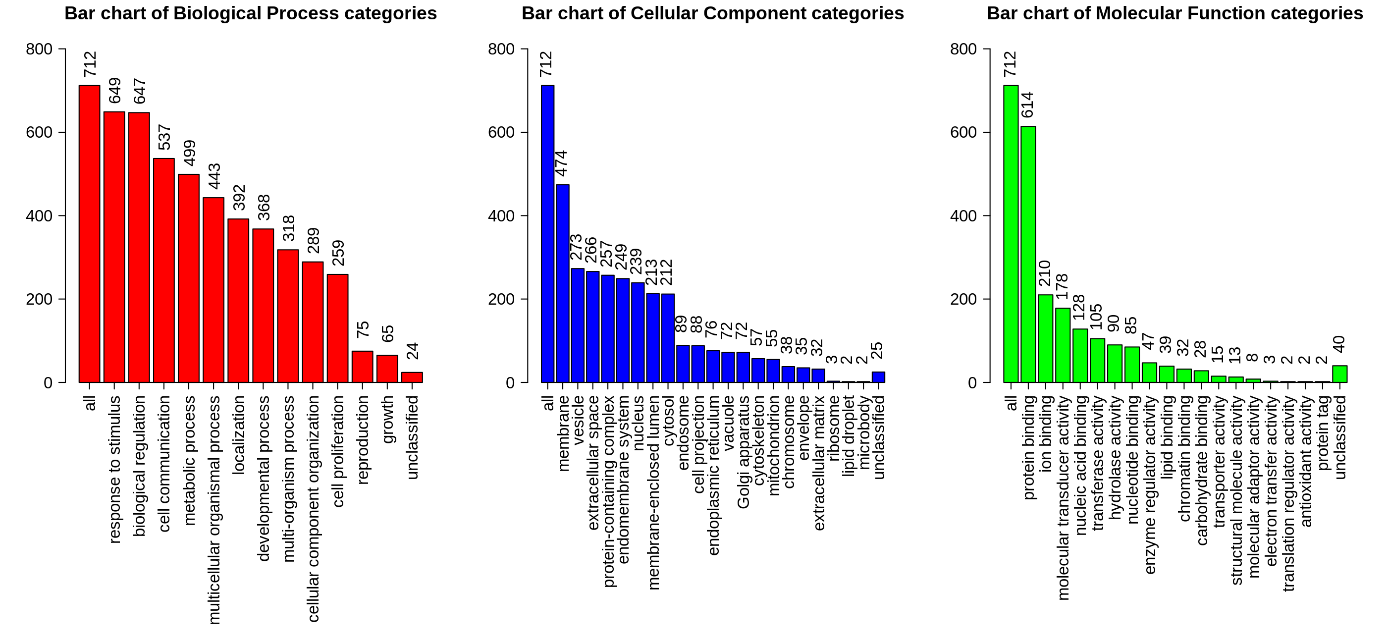


Suppl. Figure 6: To gain further insight into which biological processing are affected by differential gene expression in patients showing signs of altered epitope processing (sample cohort), a gene set enrichment analysis was performed. The gene ontology (GO) analysis is part of the greater enrichment analysis. The analysis was performed to estimate the correlation between a patient group/escape mechanism and certain biological functions (red chart), cellular components (blue chart) and molecular functions (green chart).


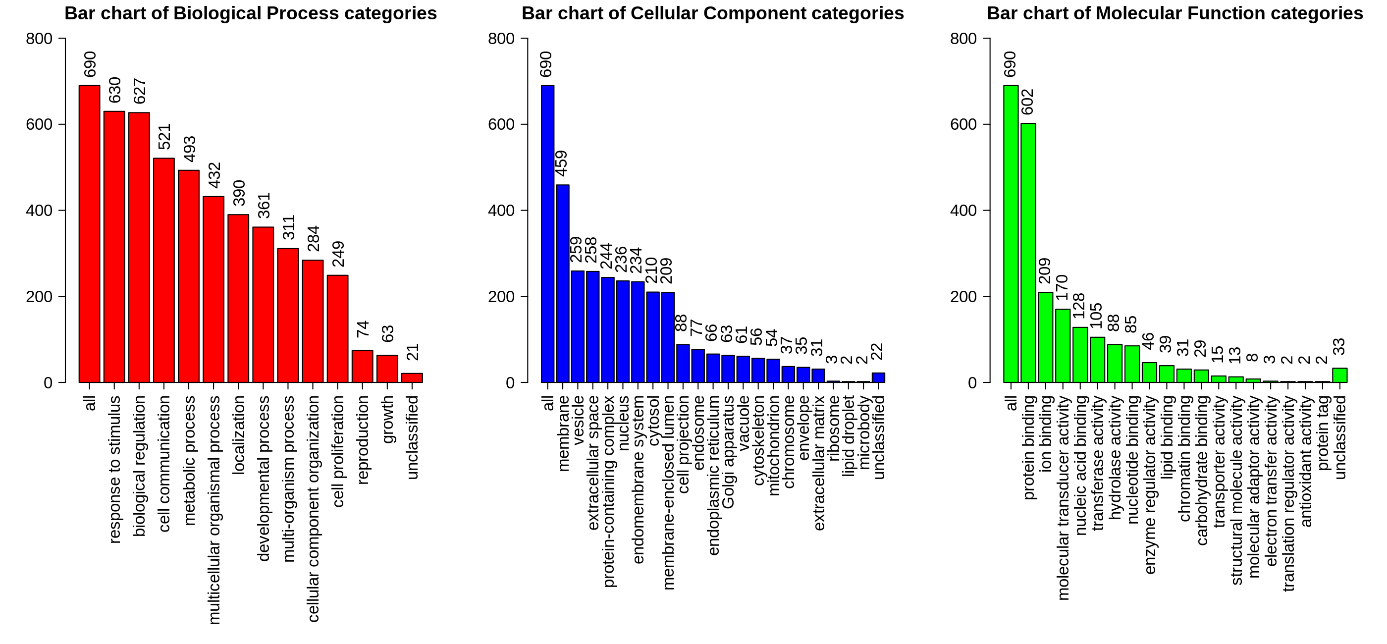


Suppl. Figure 7: To gain further insight into which biological processing are affected by differential gene expression in patients showing signs of altered epitope processing (validation cohort), a gene set enrichment analysis was performed. The gene ontology (GO) analysis is part of the greater enrichment analysis. The analysis was performed to estimate the correlation between a patient group/escape mechanism and certain biological functions (red chart), cellular components (blue chart) and molecular functions (green chart).


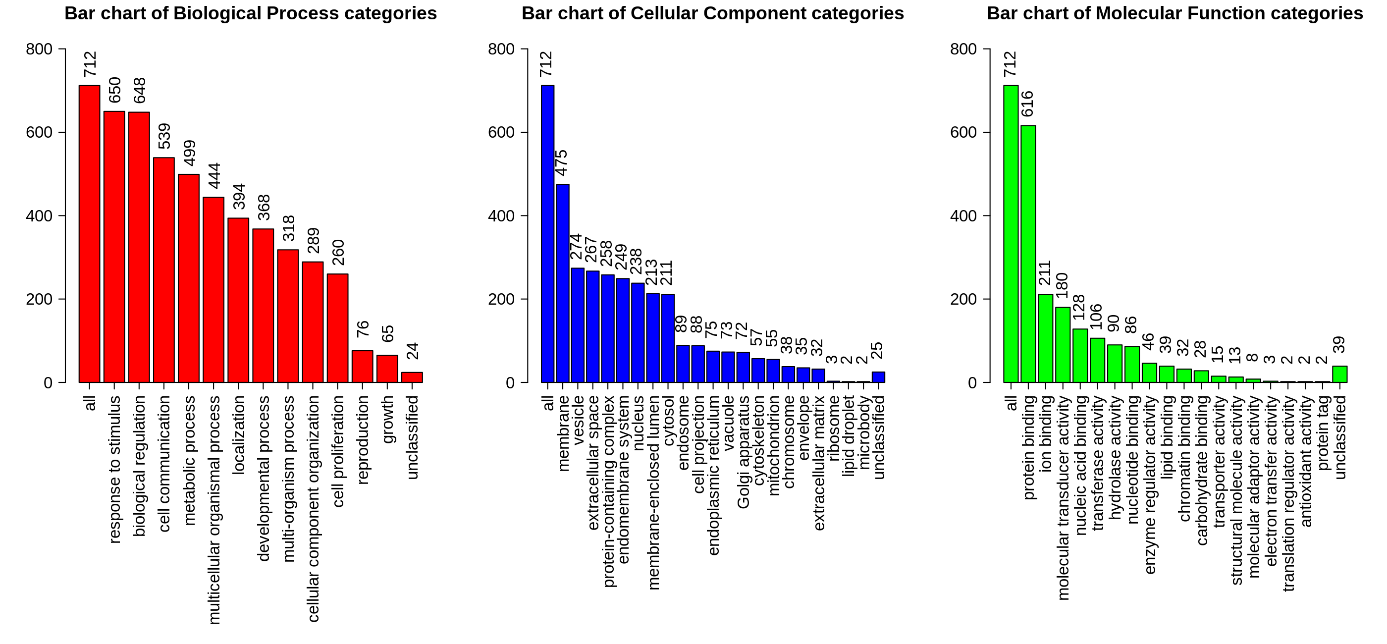


Suppl. Figure 8: To gain further insight into which biological processing are affected by differential gene expression in patients displaying both phenotypes (PD-L1 expression and altered epitope processing), a gene set enrichment analysis was performed. The gene ontology (GO) analysis is part of the greater enrichment analysis. The analysis was performed to estimate the correlation between a patient group/escape mechanism and certain biological functions (red chart), cellular components (blue chart) and molecular functions (green chart).


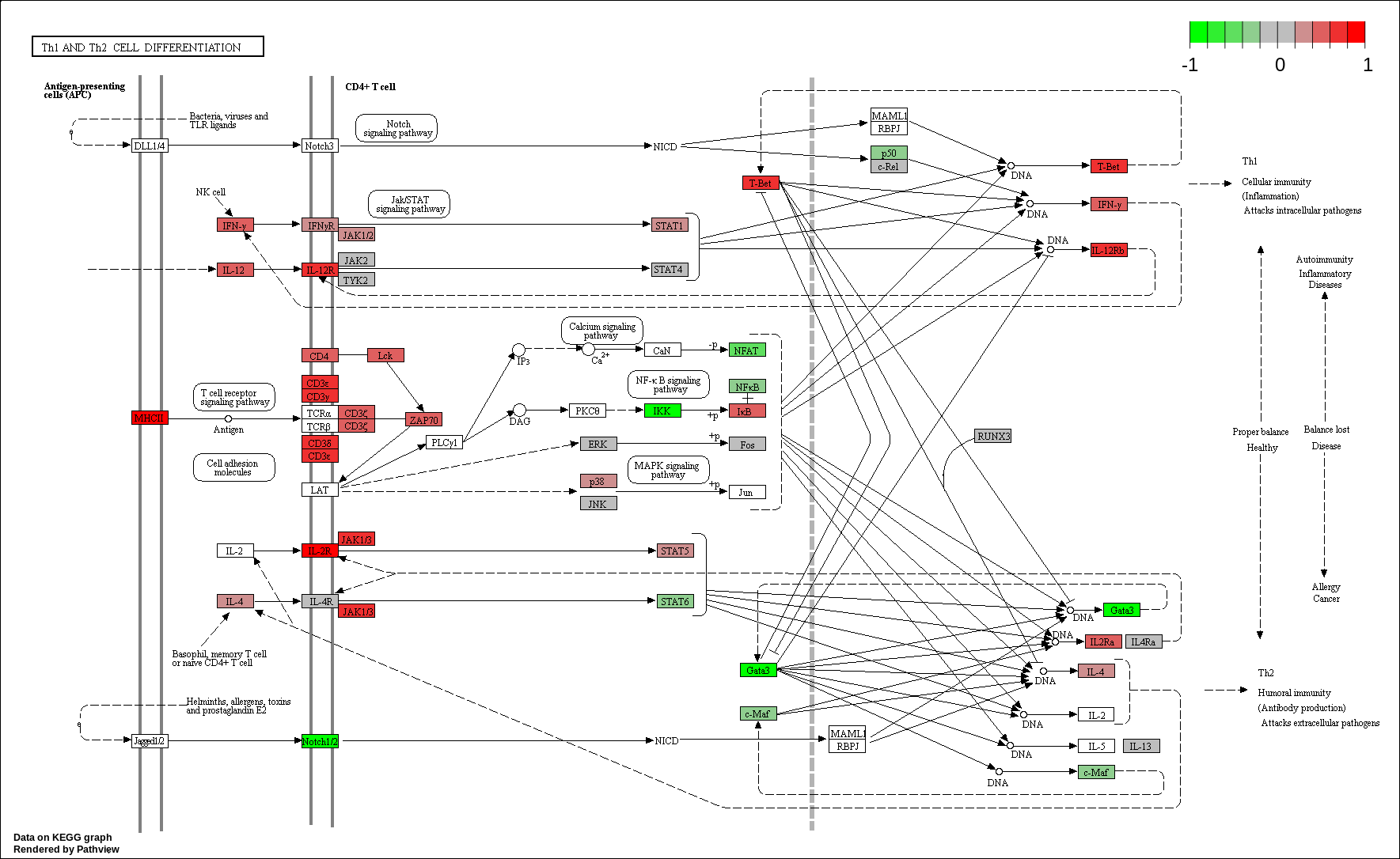


Suppl. Figure 9: KEGG pathway analysis of T helper cell (subtype 1 and 2) differentiation in patients expressing PD-L1. The plots were created via the pathview package in R. Genes are either strongly expression (red) or their expression is reduced (green).


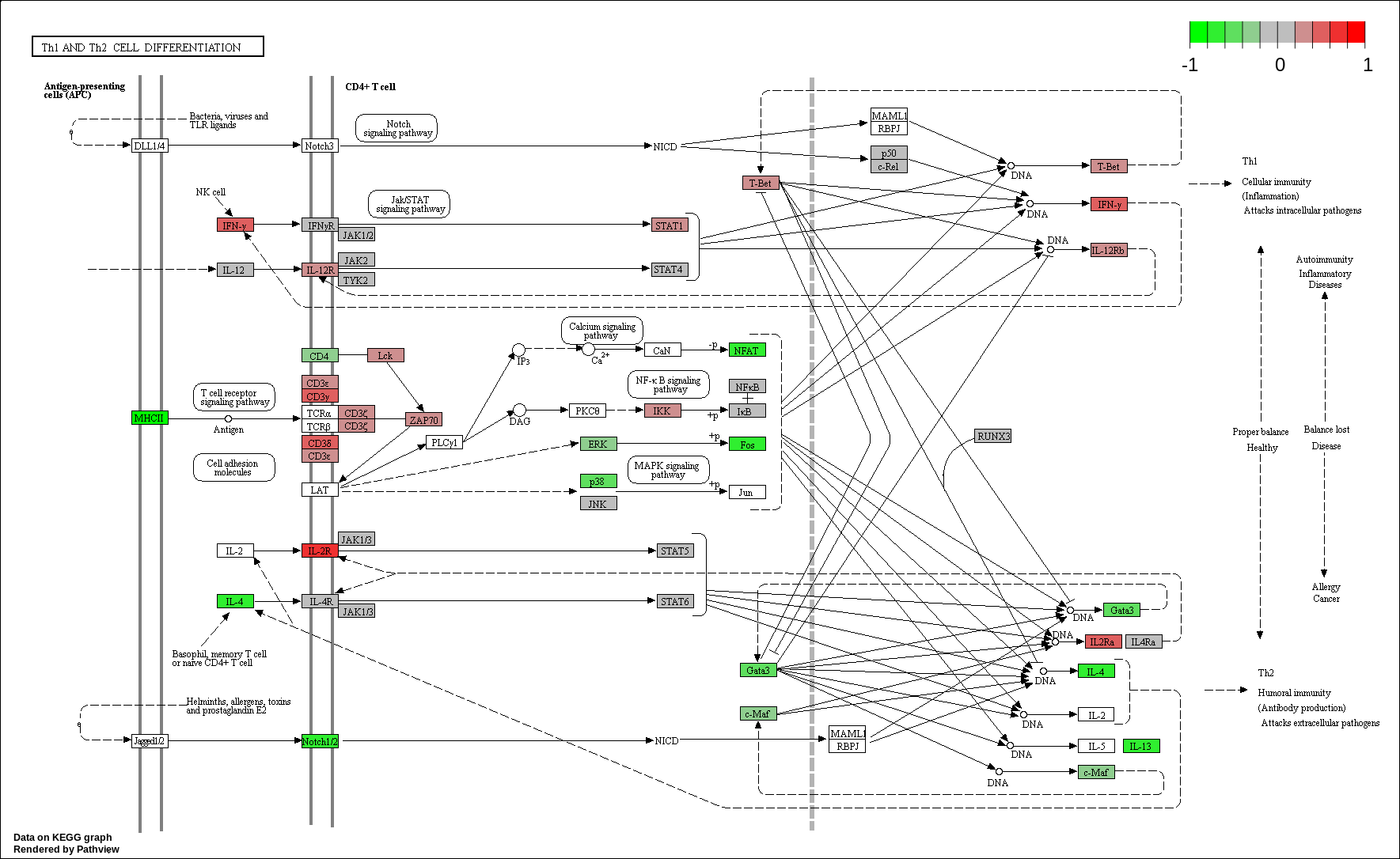


Suppl. Figure 10: KEGG pathway analysis of T helper cell (subtype 1 and 2) differentiation in patients showing signs of altered epitope processing (sample cohort). The plots were created via the pathview package in R. Genes are either strongly expression (red) or their expression is reduced (green).


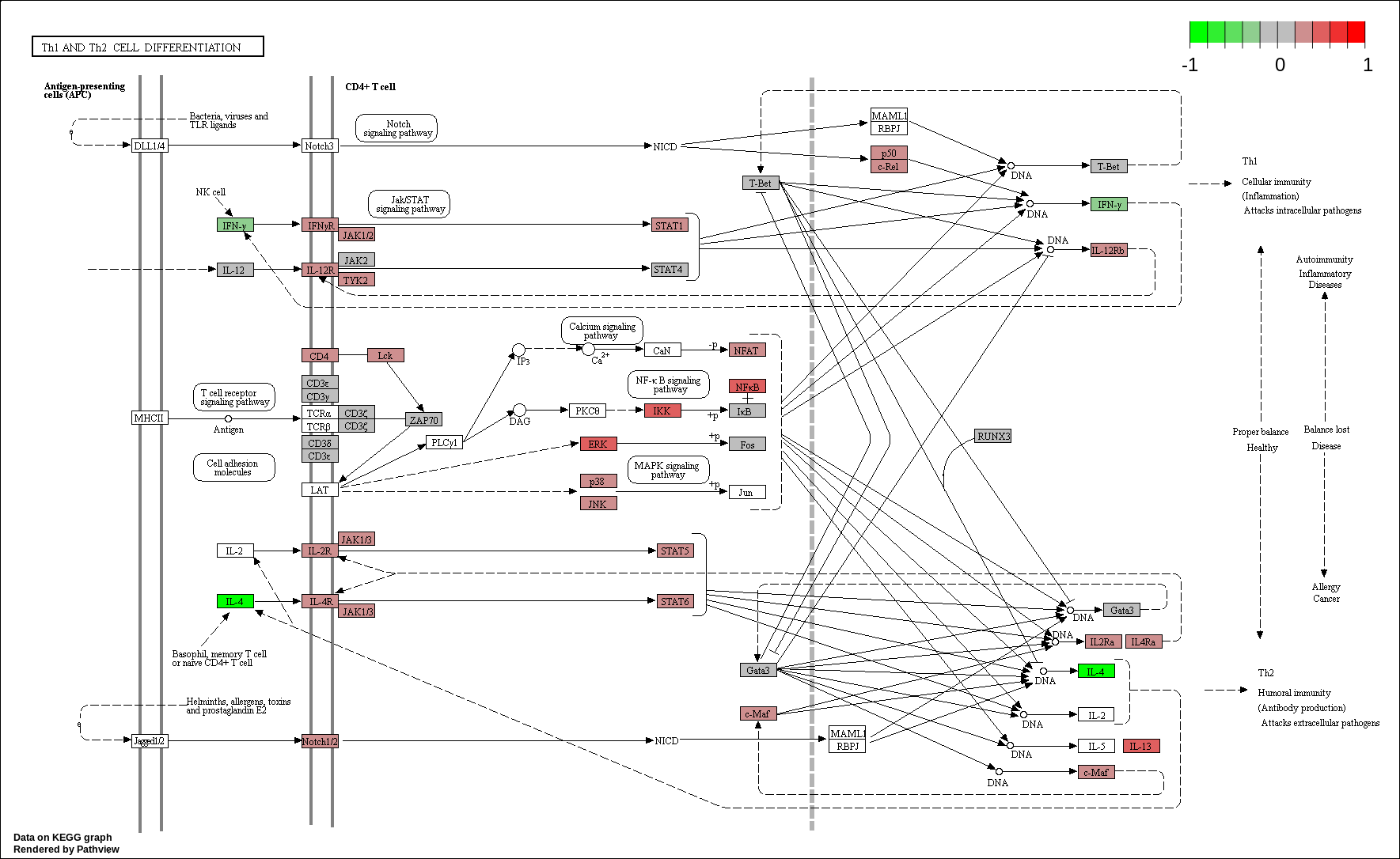


Suppl. Figure 11: KEGG pathway analysis of T helper cell (subtype 1 and 2) differentiation in patients showing signs of altered epitope processing (validation cohort). The plots were created via the pathview package in R. Genes are either strongly expression (red) or their expression is reduced (green).


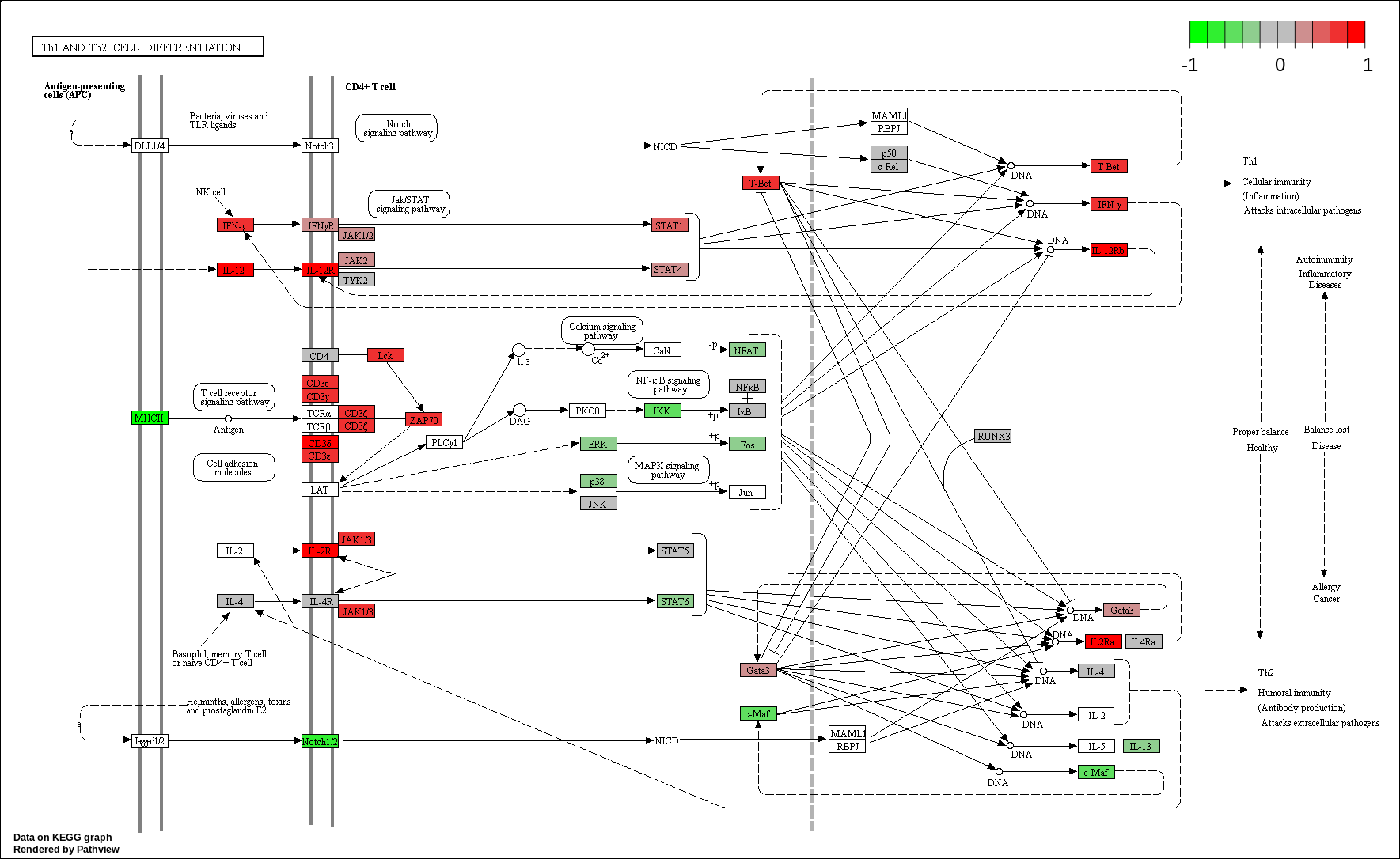


Suppl. Figure 12: KEGG pathway analysis of T helper cell (subtype 1 and 2) differentiation in patients showing both signs of altered epitope processing, but also expressing high levels of PD-L1. The plots were created via the pathview package in R. Genes are either strongly expression (red) or their expression is reduced (green).

Cytotoxic T-Cell

Cancer Cell

TCR Signaling

TCR

PD-1

PDL-1

HLA-I

Suppl. Figure 13: Activation of cytotoxic lymphocytes by tumour neoepitopes under immune checkpoint therapy. The figure additionally highlights the role of altered processing during the process. Through high mutational load, mutations change proteasomal cleavage patterns leading to structural changes or disruption of the original epitope. Regardless, the immunogenicity of the tumour neoepitopes is lowered. The T cell does not become active and immune checkpoint inhibition is rendered ineffective, since it cannot promote weak or absent signaling.


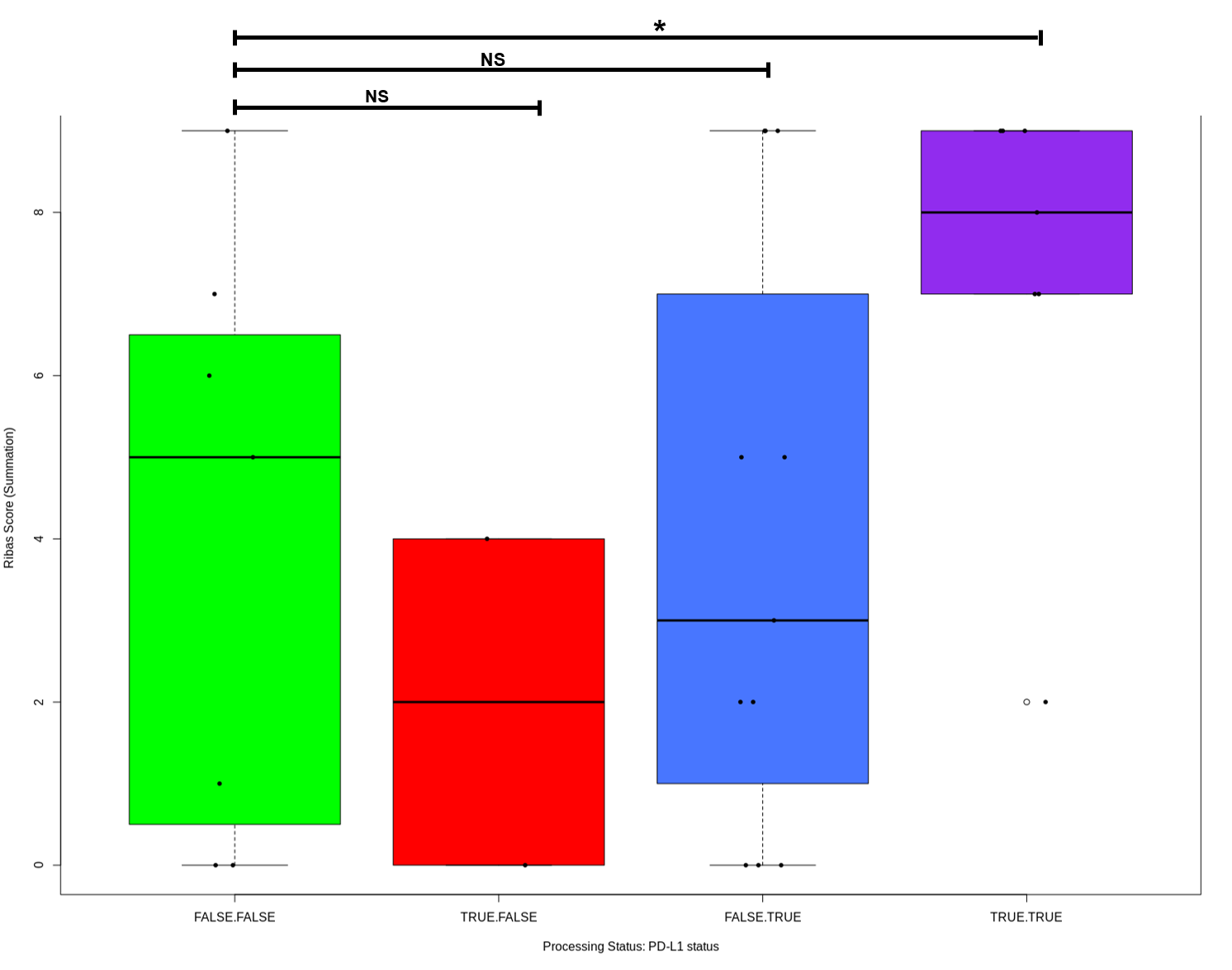


Suppl. Figure 14: Expression of IFN gamma associated genes* in association with patients expressing PD-L1 or showing signs of altered processing. FALSE/FALSE: Patients displaying neither group (green), TRUE/FALSE: Patients display signs of altered epitope processing, but no signs of PD-L1 expression (red). FALSE/TRUE: Patients displaying signs of PD-L1 expression, but without any signs of altered epitope processing (blue). TRUE/TRUE: Patients show signs of both mechanisms (violet). NS: Not significant. * p= 0.05926.


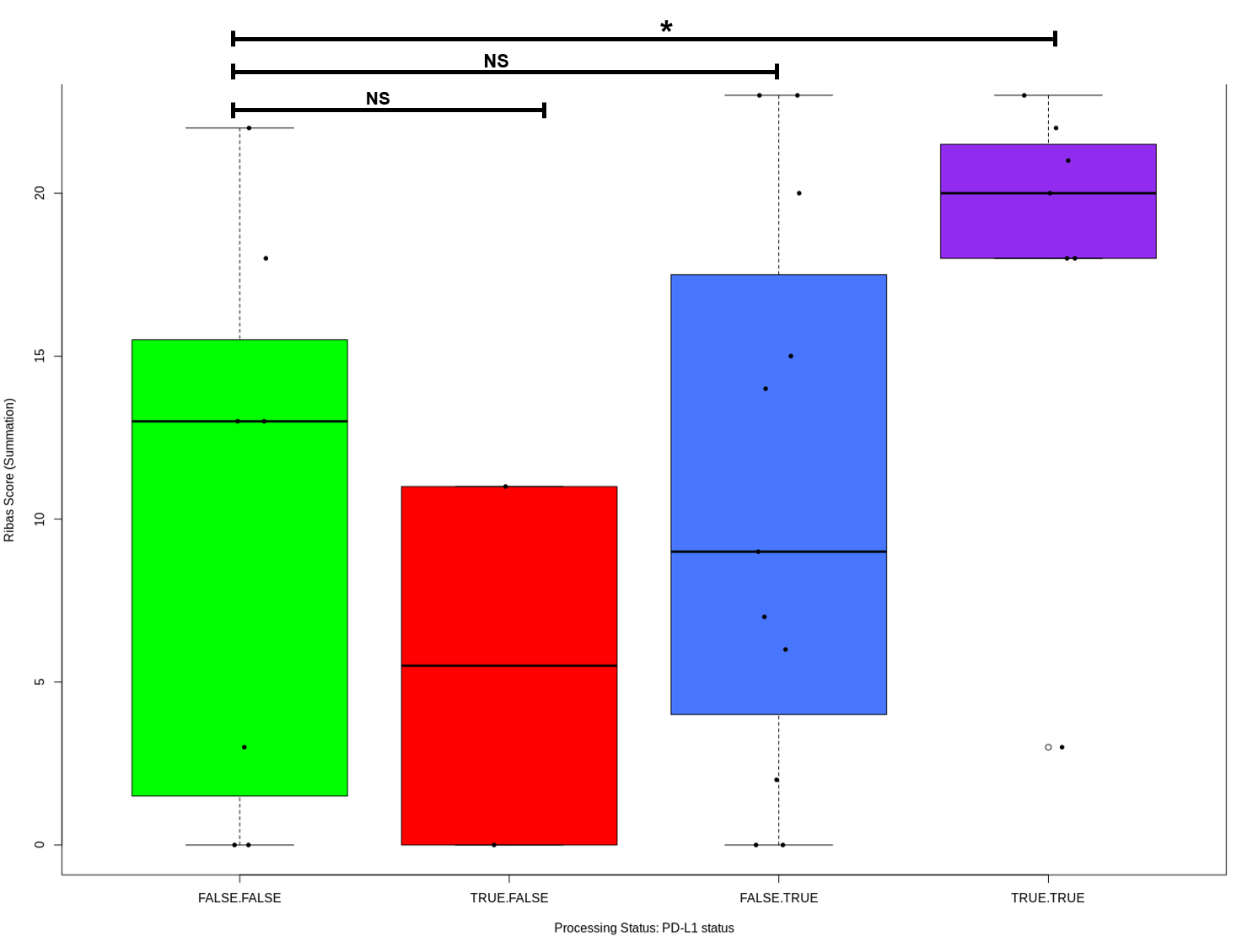


Suppl. Figure 15: Expression of an extended mRNA based immune signature* in association with patients expressing PD-L1 or showing signs of altered processing. FALSE/FALSE: Patients displaying neither group (green), TRUE/FALSE: Patients display signs of altered epitope processing, but no signs of PD-L1 expression (red). FALSE/TRUE: Patients displaying signs of PD-L1 expression, but without any signs of altered epitope processing (blue). TRUE/TRUE: Patients show signs of both mechanisms (violet). NS: Not significant. * p= 0.05623.

* IFN gamma and immune scoring:

According to Ayers et al 2017 it is possible to infer to immune activity based on the expression of distinct key genes. Those key genes are either associated with IFNγ (Interferon gamma) expression specifically or the larger overall immune response. Regression analysis was performed for every gene described by Ayers et al. 2017 for both the IFN gamma signature and the expanded immune gene signature. The final score was calculated by forming the sum across all results from the regression analysis.


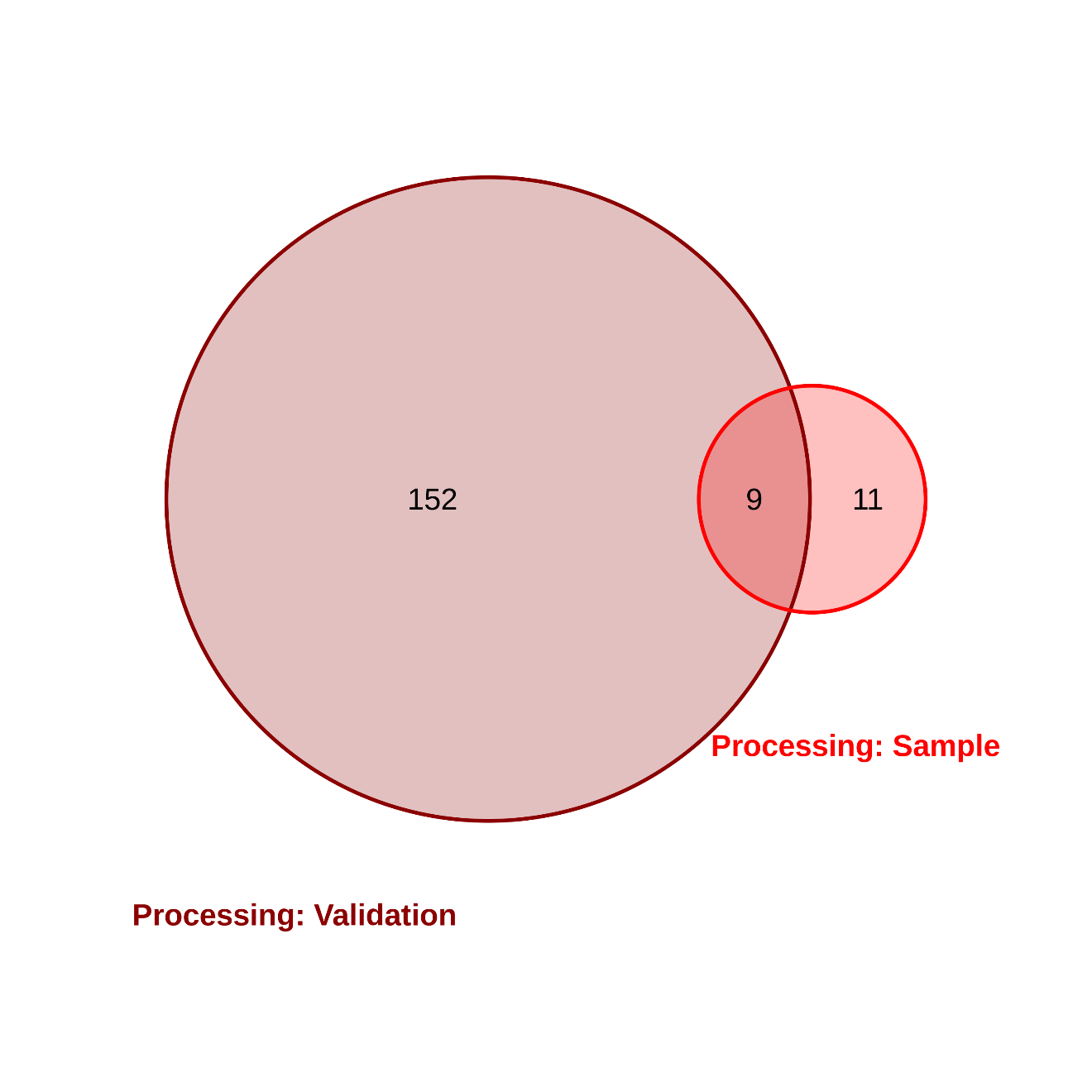


Suppl. Figure 16: The discovery cohort (“Sample”, red) and the validation cohort (dark red) were compared regarding gene expression in association with altered epitope processing.
